# Transient proteolysis reduction of *Nicotiana benthamiana*-produced CAP256 broadly neutralizing antibodies using CRISPR/Cas9

**DOI:** 10.3389/fpls.2022.953654

**Published:** 2022-08-18

**Authors:** Advaita Acarya Singh, Priyen Pillay, Previn Naicker, Kabamba Alexandre, Kanyane Malatji, Lukas Mach, Herta Steinkellner, Juan Vorster, Rachel Chikwamba, Tsepo L. Tsekoa

**Affiliations:** ^1^Future Production: Chemicals Cluster, Council for Scientific and Industrial Research, Pretoria, South Africa; ^2^Department of Plant and Soil Sciences, University of Pretoria, Pretoria, South Africa; ^3^NextGen Health Cluster, Council for Scientific and Industrial Research, Pretoria, South Africa; ^4^Department of Applied Genetics and Cell Biology, University of Natural Resources and Life Sciences, Vienna, Austria

**Keywords:** plant biotechnology, CRISPR/Cas9, genome editing, *Nicotiana benthamiana*, proteases, immunoglobulin G, human immunodeficiency virus

## Abstract

The hypersensitive response is elicited by *Agrobacterium* infiltration of *Nicotiana benthamiana*, including the induction and accumulation of pathogenesis-related proteins, such as proteases. This includes the induction of the expression of several cysteine proteases from the C1 (papain-like cysteine protease) and C13 (legumain-like cysteine protease) families. This study demonstrates the role of cysteine proteases: *Nb*VPE-1a, *Nb*VPE-1b, and *Nb*CysP6 in the proteolytic degradation of *Nicotiana benthamiana* (glycosylation mutant ΔXTFT)-produced anti-human immunodeficiency virus broadly neutralizing antibody, CAP256-VRC26.25. Three putative cysteine protease cleavage sites were identified in the fragment crystallizable region. We further demonstrate the transient coexpression of CAP256-VRC26.25 with CRISPR/Cas9-mediated genome editing vectors targeting the *NbVPE-1a, NbVPE-1b*, and *NbCysP6* genes which resulted in a decrease in CAP256-VRC26.25 degradation. No differences in structural features were observed between the human embryonic kidney 293 (HEK293)-produced and ΔXTFT broadly neutralizing antibodies produced with and without the coexpression of genome-editing vectors. Furthermore, despite the presence of proteolytically degraded fragments of plant-produced CAP256-VRC26.25 without the coexpression of genome editing vectors, no influence on the *in vitro* functional activity was detected. Collectively, we demonstrate an innovative *in planta* strategy for improving the quality of the CAP256 antibodies through the transient expression of the CRISPR/Cas9 vectors.

## Introduction

Broadly, neutralizing antibodies (bNAbs) are antibodies with the ability to neutralize multiple HIV-1 strains by targeting conserved epitopes of the virus (Burton and Hangartner, 2016). Antibody neutralization is exclusively targeted to the Env, due to it being the only surface-exposed virus-encoded protein (Davenport et al., 2011). One such antibody lineage is the CAP256-VRC26 antibodies, which are highly potent bNAb against HIV-1 subtypes A and C strains (Bhiman et al., 2015; Doria-Rose et al., 2015). The CAP256-VRC26 bNAbs lineage, like PG9, targets the trimeric variable regions 1 and 2 (V1V2) region of the HIV-1 gp120 envelope glycoprotein (McLellan et al., 2011; Doria-Rose et al., 2014; Bhiman et al., 2015). Transient plant-based production of the CAP256-VRC26.08 and CAP256-VRC26.09, along with numerous other anti-HIV antibodies (Abs), have been demonstrated in *Nicotiana benthamiana* (*N. benthamiana*) (Loos et al., 2015; Castilho et al., 2018; Stelter et al., 2019; Singh et al., 2020). *Nicotiana* species allow for the modulation of posttranslational modifications (PTMs), such as glycosylation and sulfation, which are critical to the functioning of these antibodies (Strasser et al., 2008, 2009; Loos et al., 2015; Singh et al., 2020).

A major challenge encountered with *Nicotiana* species is the *in planta* proteolytic degradation of some recombinantly produced proteins (Doran, 2006; Benchabane et al., 2008). The hypersensitive response is elicited in *N. benthamiana* in response to the technique of *Agrobacterium* infiltration. This includes the induction and accumulation of pathogenesis-related (PR) proteins, such as proteases (Goulet et al., 2010; Pitzschke, 2013; Zhou et al., 2017). The hypersensitive response not only reduces subsequent infection but may also hinder *Agrobacterium*-mediated transgene delivery, causes proteolytic degradation of recombinantly produced protein, and triggers senescence (Robinette and Matthysse, 1990; Hörtensteiner and Feller, 2002; Rico et al., 2010; Sheikh et al., 2014; Li et al., 2017). Among the induced PR proteins are several cysteine proteases, in particular, proteases from the cysteine protease families: C1 (papain-like cysteine protease, PLCP) and C13 (legumain-like cysteine protease, LLCP) (Pillay et al., 2016). Vacuolar processing enzymes (VPEs), *Nb*VPE-1a and *Nb*VPE-1b from the C13 family and *Nb*CysP6, and a *N. benthamiana* ortholog to the *Arabidopsis* drought-induced cysteine protease Responsive-to-Desiccation-21 (RD21) from the C1 family were shown to be highly upregulated post agroinfiltration (Richau et al., 2012; Pillay et al., 2016; Paireder et al., 2017; Grosse-Holz et al., 2018; Vorster et al., 2019).

The inclusion of PTMs on Abs requires transition through the secretory pathway to either the cytosol, vacuole, or apoplast; of which all these environments are enriched with either LLCPs or PLCPs, or both (Hara-Nishimura et al., 2005; Goulet et al., 2012; Hatsugai et al., 2015). Apart from the physiological roles of these LLCPs and PLCPs, these protease families have also been implicated in the degradation of plant-produced anti-HIV antibodies (Niemer et al., 2014; Hehle et al., 2015; Paireder et al., 2017). Proteolytic degradation is not limited to *in planta* degradation during protein expression, *ex planta* is also possible during extraction and downstream processing (Rivard et al., 2006; Benchabane et al., 2008; Mandal et al., 2014; Niemer et al., 2014; Hehle et al., 2015). The reduction of protease activity could potentially improve both the quality and yield of the produced recombinant protein (Paireder et al., 2017). It had been demonstrated that the downregulation of the PLCP, CysP6, in *Nicotiana tabacum* (*N. tabacum*) had a beneficial effect on the accumulation of recombinantly produced protein (Duwadi et al., 2015). VPEs are intricately linked with the level of activity of PLCPs as some VPEs are responsible for the activation of some PLCPs (Okamoto and Minamikawa, 1999; Roberts et al., 2012; Cilliers et al., 2018).

This study demonstrated the transient production of CAP256-VRC26.25, produced in the glycoengineered *N. benthamiana* (ΔXTFT) with human tyrosyl protein sulfotransferase 1 (hTPST1), as was demonstrated with the CAP256-VRC26.08 and CAP256-VRC26.09 bNAbs (Singh et al., 2020). A similar proteolytic degradation pattern for the *N. benthamiana* (ΔXTFT) produced CAP256-VRC26.25, as was previously observed with *N. benthamiana* (ΔXTFT) produced CAP256-VRC26.08 and CAP256-VRC26.09 (Singh et al., 2020). Through the simultaneous targeted transient disruption of NbCysP6, NbVPE-1a and NbVPE-1b, using clustered regularly interspaced short palindromic repeats/CRISPR associated protein 9 (CRISPR/Cas9), we demonstrate a reduction in the *in planta* protease degradation of CAP256-VRC26.25 produced in *N. benthamiana* (ΔXTFT). Our data also revealed that there were no structural and efficacy difference between the CAP256-VRC26.25 bNAbs produced with and without protease disruption.

## Materials and methods

### CAP256-VRC26.25 cloning

CAP256-VRC26.25 constructs were prepared as briefly outlined below. CAP256-VRC26.25 variable regions sequences were sourced from Bhiman, Doria-Rose, and co-workers (Bhiman et al., 2015; Doria-Rose et al., 2015) and GenBank [KT371100.1 (protein ID: ALG00386.1)] and KT371101.1 [protein ID: ALG00387.1)]. The light (LC) and heavy chains (HC) were synthesized and fused to human immunoglobulin G 1 (IgG1) lambda and gamma constant regions and each to a murine IgG signal peptide (SP). Genes were inserted in pICH31180 and pICH21161 MagnICON vectors (ICON Genetics and Nomad Bioscience, DE).

### *In planta* production of sulfated CAP256-VRC26.25

Sulfated CAP256-VRC26.25 was produced using the *Agrobacterium*-mediated transient expression system as outlined by Singh et al. (2020) through hTPST1 coexpression (Loos et al., 2015). Syringe agro-infiltration, as described by Marillonnet and co-authors, was used (Marillonnet et al., 2004). Expression vectors cloned with light and heavy chain inserts were transformed into the *Agrobacterium tumefaciens* (*A*. *tumefaciens*) strain LBA4404 (Invitrogen, MA, USA), whereas the hTPST1 vector was transformed into the *A*. *tumefaciens* strain GV3101::pMP90. *A. tumefaciens* containing the IgG and hTPST1 constructs were incubated in Luria Broth (LB) containing 50 μg.ml^−1^ kanamycin (Kan_50_) and 25 μg.ml^−1^ rifampicin (Rif_25_). *A. tumefaciens* cultures were pelleted and resuspended in infiltration buffer (10 mM MES, 10 mM MgSO_4_, and 60 mM sucrose pH 5.5) and diluted and mixed in the optimal ratios, as previously described by Singh et al. (2020). *N. benthamiana* (ΔXTFT) (Strasser et al., 2008) leaves (4–5 weeks of age) were infiltrated and leaf discs were sampled for analysis over 11 days post-infiltration (dpi). Infiltrated leaf discs were taken for each dpi and homogenized an Eppendorf^®^ micropestle (Sigma-Aldrich, USA) in the presence of.5-mm glass beads (Scientific Industries, Inc., USA), and proteins were extracted in phosphate-buffered saline (PBS) (1.5 mM KH_2_PO_4_, 8.1 mM NaHPO_4_, 2.7 mM KCl and 140 mM NaCl, pH 7.4) containing complete™ ULTRA tablets mini, EASYpack protease inhibitor cocktail (Roche, CH). Centrifugally clarified leaf extracts were separated and analyzed using 12% (w/v) polyacrylamide gel under reducing conditions, followed by Coomassie Brilliant Blue staining, Western blot analysis using goat anti-Human IgG (Fc specific)-peroxidase antibody (A0170, Sigma-Aldrich Co., USA), and goat anti-Human Lambda light chain (bound and free)-peroxidase antibody (A5175, Sigma-Aldrich Co., USA). Detection was done using the Clarity^TM^ western ECL detection kit (Bio-Rad, USA).

### *In silico* protease cleavage site analysis

Proteolytic cleavage analysis was conducted as previously described by Pillay et al. (2016). Briefly, proteolytic cleavage analysis for cysteine protease families, such as C1, C13, and C14, was conducted *in silico* using CLC Main Workbench 7.7 (http://www.clcbio.com) based on the various protease substrate specificities (Mathieu et al., 2002; Choe et al., 2006) and on the Schechter and Berger subsite nomenclature (Schechter and Berger, 1967, 1968).

### Cleavage site identification

Cleavage site identification was done by in-gel digestion as preparation for liquid chromatography with tandem mass spectrometry (LC-MS/MS). Briefly, Coomassie Brilliant Blue-stained protein bands were excised from the SDS-PAGE gel, and fragments were eluted with 50% (v/v) acetonitrile/5% (v/v) formic acid (Sigma-Aldrich, USA), following dithiothreitol (Fermentas, DE) reduction, iodoacetamide (Sigma-Aldrich USA) *S*-alkylation, and trypsin (Promega, USA) digestion. An Acclaim PepMap C18 trap (75 μm x 2 cm) column (Thermo Fischer Scientific, MA, USA) and Acclaim PepMap C18 RSLC column (75 μm x 15 cm) (Thermo Fischer Scientific, MA, USA) were used to desalt and separate peptides using a 4–60% (v/v) gradient of 80% (v/v) acetonitrile/0.1% (v/v) formic acid. Peptides were analyzed using an AB Sciex (Miami, USA) 6600 TripleTOF MS, a triple Quadrupole Time of Flight (QTOF) Mass Spectrometer (MS). MS/MS scans were in the m/z range of 100–1,800 Da. The data analysis was done using Protein Pilot (SCIEX, Canada) and Peaks v6 (Zhang et al., 2012).

### The sgRNA cloning

The CRISP-P 2.0 tool (http://crispr.hzau.edu.cn/cgi-bin/CRISPR2/SCORE) was used to design the sgRNAs (Reisch and Prather, 2015, 2017). The sgRNA:*Nb*CysP6 targeted the *Nb*CysP6 gene (KX375796.1) between positions 661…680, whereas sgRNA:*Nb*VPE-1a/b targeted both the *Nb*VPE-1a (AB181187.1) and *Nb*VPE-1b genes (AB181188.1) between positions 665…684. Genome editing vectors, pICH86966::AtU6p::sgRNA_PDS and pK7WGF2::hCas9, which were used in this study were previously reported by Nekrasov et al. (2013). sgRNAs were inserted into pICH86966::AtU6p::sgRNA_PDS through circular polymerase extension cloning (CPEC). Vectors were transformed into *E. coli* DH10β and sequenced by Inqaba Biotechnical Industries (Pty) Ltd (ZA) using sequencing primers listed in Supplementary Table 2. Plasmids were deposited in Addgene under the following names and IDs:pICH86966::AtU6p::sgRNA: NbCysP6 (223217) and pICH86966::AtU6p::sgRNA: NbVPE1a/b (223218).

### Coexpression of genome editing vectors with CAP256-VRC26.25

pICH86966::AtU6p::sgRNA:*Nb*CysP6, pICH86966::AtU6p::sgRNA:*Nb*VPE1a/b, and pK7WGF2::hCas9 vectors were transformed into the *A. tumefaciens* strain AGL1, as previously described by Nekrasov et al. (2013). *A. tumefaciens* containing the CAP256-VRC26.25 subunits, hTPST1, and genome editing constructs were cultured in LB containing 50 μg.ml^−1^ kanamycin (Kan_50_) and 25 μg.ml^−1^ rifampicin (Rif_25_). *A. tumefaciens* cultures were pelleted, resuspended, and diluted to optimal ratios, as previously described by Singh et al. (2020), with the addition of Cas9 and the sgRNA at an optical density (OD) of 1 in various sgRNA combinations to create various infiltration mixtures (Supplementary Table 3) to best facilitate the reduction of CAP256-VRC26.25 proteolytic degradation. *N. benthamiana* (ΔXTFT) (Strasser et al., 2008) leaves (4–5 weeks of age) were infiltrated and leaf discs were sampled for analysis at 7 dpi. Infiltrated leaf discs were homogenized and centrifugally clarified, with leaf extracts separated and analyzed using 12% (w/v) polyacrylamide gel under reducing condition, followed by Coomassie Brilliant Blue staining and Western blot analysis as aforementioned to determine the optimal combination for proteolytic degradation reduction. The optimal combination was further analyzed over a period of 11 dpi using a 12% (w/v) polyacrylamide gel and Western blot.

### T7 endonuclease I based confirmation of genome editing

Confirmation of CRISPR/Cas9-mediated disruption of the targeted cysteine protease genes was determined using the T7 endonuclease I assay. The assay was adapted from the experimental procedure outlined by Guschin et al. (2010). T7 endonuclease I recognizes and cleaves at Holliday junctions in an annealed PCR product (Vouillot et al., 2015). Samples from CAP256-VRC26.25 and hTPST1 expression, combined with or without the disruption of *Nb*CysP6 and/or *Nb*VPE-1a and *Nb*VPE-1b (Supplementary Table 3), were assessed using the T7 endonuclease I assay. Plant genomic DNA was extracted from 200 mg of leaf discs using a DNeasy^®^ plant mini kit (Qiagen, DE), as per the manufacturer's guidelines. Polymerase chain reaction (PCR) primers (Supplementary Table 4) were designed to amplify fragments of the *Nb*CysP6, *Nb*VPE-1a and *Nb*VPE-1b genes, with 250 base pairs (bps) spanning each side of the CRISPR/Cas9 disruption site of the protease genes, which results in a ~520 bp amplicon. The primer sets were used in separate PCR amplifications of the respective gene targets in the uninfiltrated samples and samples where both sgRNA:*Nb*CysP6 and sgRNA:*Nb*VPE1a/b were utilized. The PCR was set up for the amplification of regions within the *Nb*CysP6, *Nb*VPE-1a, and *Nb*VPE-1b genes using Q5^®^ Hot Start High-Fidelity 2X master mix (New England Biolabs, USA), as per the manufacturer's instruction. PCR amplicons were purified using the GeneJET PCR purification kit (Thermo Fisher Scientific, USA), as per the manufacturer's guidelines. The T7 endonuclease I (New England Biolabs, USA) was used as outlined by the manufacturers. Post-hybridization step and T7 endonuclease I digestion, DNA fragments were purified using the GeneJET PCR purification kit, as per the manufacturer's guidelines. Digested DNA fragments were separated on a 5% *tris*(hydroxymethyl)aminomethane/boric acid/ethylenediaminetetraacetic acid (TBE) acrylamide gel, and visualized using GelRed (Biotium, USA).

### RT-qPCR analysis of candidate cysteine protease mRNA fluctuations

mRNA transcripts for three proteolytic candidate proteases (*Nb*CysP6, *Nb*VPE1a, and *Nb*VPE1b) were assayed on a CFX96 real-time PCR detection system (Bio-Rad, USA) by Inqaba Biotechnical Industries (Pty) Ltd (ZA). Total RNA was extracted from 150 mg of leaf discs using the Zymo Quick RNA Plant kit (Zymo Research, USA), as per the manufacturer's instructions. RNA concentration was determined using the NanoDrop™ One Microvolume UV-Vis Spectrophotometer (Thermo Fisher Scientific, USA). cDNA synthesis was done using the LunaScript^®^ RT SuperMix kit (New England Biolabs, USA), according to the manufacturer's instructions, except it was done in a total volume of 20 μl containing 500 ng total RNA. Quantitative RT-PCR was then performed in 96-well-pplates with the Luna^®^ Universal qPCR Master Mix (New England Biolabs, USA) using a dye-based qPCR assay. Each reaction contained 1 μl of cDNA template, 0.25 μm of forward and reverse primers (Supplementary Table 5), and 1X Luna^®^ Universal qPCR Master mix. Reactions were analyzed using a standard two-step PCR program, as described in the Luna Universal qPCR Master Mix manual. Each cDNA was analyzed in triplicates. Amplification of different input templates was evaluated based on the quantification cycle (Cq) value. Fold changes in gene expression were determined using the Livak method (Livak and Schmittgen, 2001). For comparative purposes, relative gene expression levels of the candidate proteases were defined with the value of 1 set for control uninfiltrated plants and normalized against relevant reference genes, as previously described by Pillay et al. (2016).

### Enzyme-linked immunosorbent assay

Abs produced in the presence and absence of genome editing vectors were quantified by sandwich enzyme-linked immunosorbent assay (ELISA) over a period of 11 dpi. A total of 96-well-plates were coated with either 5 μg/ml of goat anti-human lambda LC (bound and free) antibody (L1645, Sigma-Aldrich, USA) or goat anti-human IgG (Fc specific) antibody (I2136, Sigma-Aldrich, USA). All wells containing capture-antibody were blocked for 16 h at 4°C with 5% fat-free milk in PBS, pH 7.4. After washing the wells with PBS, pH 7.4 containing 0.1% (w/v) Tween^®^-20, a standard concentration range was prepared using purified IgG1 (I5029, Sigma-Aldrich, St. Louis, USA), and clarified leaf extracts containing Abs produced in the presence and absence of genome editing vectors were incubated with the respective capture antibody for 16 h at 4°C. This was followed by wells being washed and bound IgGs being detected using either goat anti-Human IgG (Fc specific)-peroxidase antibody (A0170, Sigma-Aldrich, USA) or goat anti-Human Lambda light chain (bound and free)-peroxidase antibody (A5175, Sigma-Aldrich, USA), which were incubated with sample wells for 2 h at 37°C, followed by a wash. The peroxidase substrate, 3,3',5,5'-Tetramethylbenzidine (TMB) substrate solution (Sigma-Aldrich, USA) was used, with peroxidase-TMB reactions being stopped with 1 M H_2_SO_4_, and readings taken using a Hidex Sense Microplate Reader fluorometer (Hidex, EE) at 450 nm.

### Cathepsin L-like protease activity assay

TSP from 25 mg of leaf disc was extracted in Cathepsin L assay buffer (50 mM Na_2_HPO_4_ (Merck KGaA, DE) and 10 mM L-cysteine (Sigma-Aldrich, DE), pH 6.0). Clarified leaf extracts were transferred into black, flat-bottom polysorp 96-well-plates (Nunc, DK). Approximately 8 μM of the Z-Phe-Arg-7-amido-4-methylcoumarin hydrochloride (Z-Phe-Arg-MCA) (Sigma-Aldrich, DE) substrate was added to each well, and fluorescence was measured kinetically over a 10-min period. Fluorescent measurements were taken on a Hidex Sense Microplate Reader fluorometer (Hidex, EE) at 25°C with an excitation wavelength of 360 nm and an emission wavelength of 450 nm. A broad-spectrum protease inhibitor, N-[N-(L-3-transcarboxyirane-2-carbonyl)-L-Leucyl]-agmatine (E-64) (Sigma-Aldrich, DE), was used at 10-μm concentration to inhibit papain-like cysteine activity. Statistically significant changes in activity levels between samples, with or without CAP256-VRC26.25 and hTPST1 expressions, combined with or without the disruption of *Nb*CysP6 and/or *Nb*VPE-1a and *Nb*VPE-1b, were determined using ANOVA: single-factor with replication (*p* < 0.001) calculated using Microsoft Excel software 2010 version 14 (Microsoft Corporation, USA) and *t*-tests (*p* < 0.05 and < 0.001).

### Legumain protease activity assay

TSP from 25 mg of leaf disc was extracted in Legumain assay buffer (121 mM Na_2_HPO_4_ (Merck KGaA, DE), 39.5 mm of citric acid (Sigma-Aldrich, DE), 1 mm of DL-Dithiothreitol (DTT) (Fermentas, DE), 1 mm of Na_2_EDTA (Sigma-Aldrich, DE), and.01% of CHAPS (Biorad, USA), (Sigma-Aldrich, DE), pH 5.8). Clarified leaf extracts were transferred into black, flat-bottom polysorp 96-well-plates (Nunc, DK). About 1 mm of Z-Ala-Ala-Asn-AMC (Z-AAN-AMC) (Bachem, DE) substrate was added to each well, and fluorescence was measured kinetically over a 10-min period. Fluorescent measurements were taken on a Hidex Sense Microplate Reader fluorometer (Hidex, EE) at 25°C with an excitation wavelength of 360 nm and an emission wavelength of 450 nm. E-64 (Sigma-Aldrich, DE) was used at 10 μm concentration to inhibit papain-like cysteine protease, which may also cleave the legumain substrate. Statistically significant changes in activity levels between samples, with or without CAP256-VRC26.25 and hTPST1 expressions, combined with or without the disruption of *Nb*CysP6 and/or *Nb*VPE-1a and *Nb*VPE-1b, were determined using ANOVA: single-factor with replication (*p* < 0.001) calculated using Microsoft Excel software 2010 version 14 (Microsoft Corporation, USA) and *T*-tests (*p* < 0.05 and < 0.001).

### Host total soluble protein quantification

Quantification of host total soluble protein (TSP) was done, as previously described by Buyel et al. (2016). Briefly, TSP was extracted from 200 mg of leaf discs for each sample and quantified with the Bradford method (Bradford, 1976), using Quick Start™ Bradford 1x dye reagent (Biorad, USA). A 5-μl sample was added to 250 μl of Quick Start™ Bradford 1x dye reagent, of which 200 μl was transferred into a well of a 96-well-flat-bottom plate (Nunc, DK). All samples were analyzed in triplicates. TSP concentration was determined against a bovine serum albumin (BSA) standard curve. The BSA standards were prepared by 1:1 serial dilution (10–0.625 mg/ml) of a 20-mg/ml BSA stock (New England Biolabs, USA) using PBS, and pH 7.4. TSP concentration was calculated against a BSA standard curve. A *t*-tests (*p* < 0.05 and < 0.001) were performed to determine statistical differences between samples.

### Ab purification

Briefly, infiltrated 7 dpi whole-leaf samples were homogenized using a Matstone 6-in-one juice extractor (Matstone, PH), protein were extracted using PBS, pH 7.4 containing cOmplete™ ULTRA tablets mini, and EASYpack protease inhibitor cocktail (Roche, CH). The supernatant was clarified by a series of centrifugation and filtration steps. The bNAbs were then purified by protein A affinity chromatography using a HiTrap^®^ Protein A High-Performance column (Cytiva, USA), coupled to a Äkta Avant 150 (Cytiva, USA), as per the manufacturer's instructions.

### Oligomeric and degradation analysis of the CAP256-VRC26 Abs

The use of size-exclusion chromatography (SEC) separated molecules according to their size and, therefore, enabling the detection of fully assembled, monomeric, cleaved, and aggregated CAP256-VRC26.25 produced in *N. benthamiana* (ΔXTFT), with and without the disruption of *Nb*CysP6 and/or *Nb*VPE-1a and *Nb*VPE-1b. CAP256-VRC26.25 produced in HEK293 cells, was used as a positive control. SEC was performed on an Agilent 1,100 series high-pressure liquid chromatography (HPLC) (Agilent Technologies Inc., DE), which consisted of a binary pump, degasser, well-plate autosampler (WPALS), autosampler thermostat (ALS Thermo), column thermostat compartment, and a diode-array detector (DAD). The analytical column used was an AdvanceBio SEC 300 Å 2.7 um, 7.8 x 300 mm column (Agilent Technologies Inc., DE). All data analysis was performed using Chemstation chromatography data management software, revision B.04.02 (Agilent Technologies Inc., DE). The mobile phase contained 150 mM of Na_2_HPO_4_ (Merck KGaA, DE), pH 7. The column was conditioned for 1 h with mobile phase before the injection of samples. CAP256-VRC26.25 bNAbs were eluted isocratically at a flow rate of.35 ml/min for 20 min, and the column effluent was monitored at 220 nm. All data were collected at 30°C.

### Secondary structure analysis

Far-UV Circular dichroism (CD) spectrum (260–180 nm) measurements for 4 μM of purified bNAb samples in 10 mM of Na_2_HPO_4_ (Merck, DE), pH 7.4 were taken on an Applied Photophysics Chirascan CD spectrometer (UK) using a 1-mm path length at 20°C. Averaged ellipticity values were converted to mean residue ellipticity (MRE) and corrected for the buffer blank baseline.

### Intrinsic fluorescence analysis

Approximately 4 μM of purified bNAbs samples in 10 mM of Na_2_HPO_4_ (Merck, DE), pH 7.4 was selectively excited at 280 and 295 nm using a Shimadzu RF-530K spectrofluorophotometer (Shimadzu Corp., Kyoto, Japan). Fluorescent measurements were taken from 295 to 500 nm at 20°C, using a 2-mm quartz cuvette.

### HIV-1 neutralization assay

HIV-1 neutralization potency for each bNAb sample was assessed using the TZM-bl neutralization assay, as described previously (Montefiori, 2005). Each Ab sample was diluted in a threefold dilution series using Dulbecco's Modified Eagle's medium (DMEM) (Sigma-Aldrich, USA), with 10% Fetal Bovine Serum (FBS) (Sigma-Aldrich, USA) (growth media). Diluted Ab was added in duplicate with 100 μl/ well of a 96-well-plate. About 50 μl/well of 200 TCID_50_ of HIV-1 pseudovirus was, thereafter, introduced and incubated at 37°C for 1 h. This was followed by the addition of TZM-bl cells at the concentration of 1 × 10^4^ cells/100 μl of growth medium containing 37.5 μg/ml of DEAE dextran to each well, followed by culture at 37°C for 48 h. HIV-1 neutralization was determined by measuring the luminescence emitted by the cells using the Infinite F500 plate reader (Tecan, AT). Titers were calculated as the concentration that caused 50% reduction (IC_50_) of relative light unit (RLU) compared to the virus control (wells with no bNAb) after the subtraction of the background (wells without both the virus and the bNAb).

## Results

### Transient expression of *in planta* CAP256-VRC26.25 bNAbs

**Figure 1:**
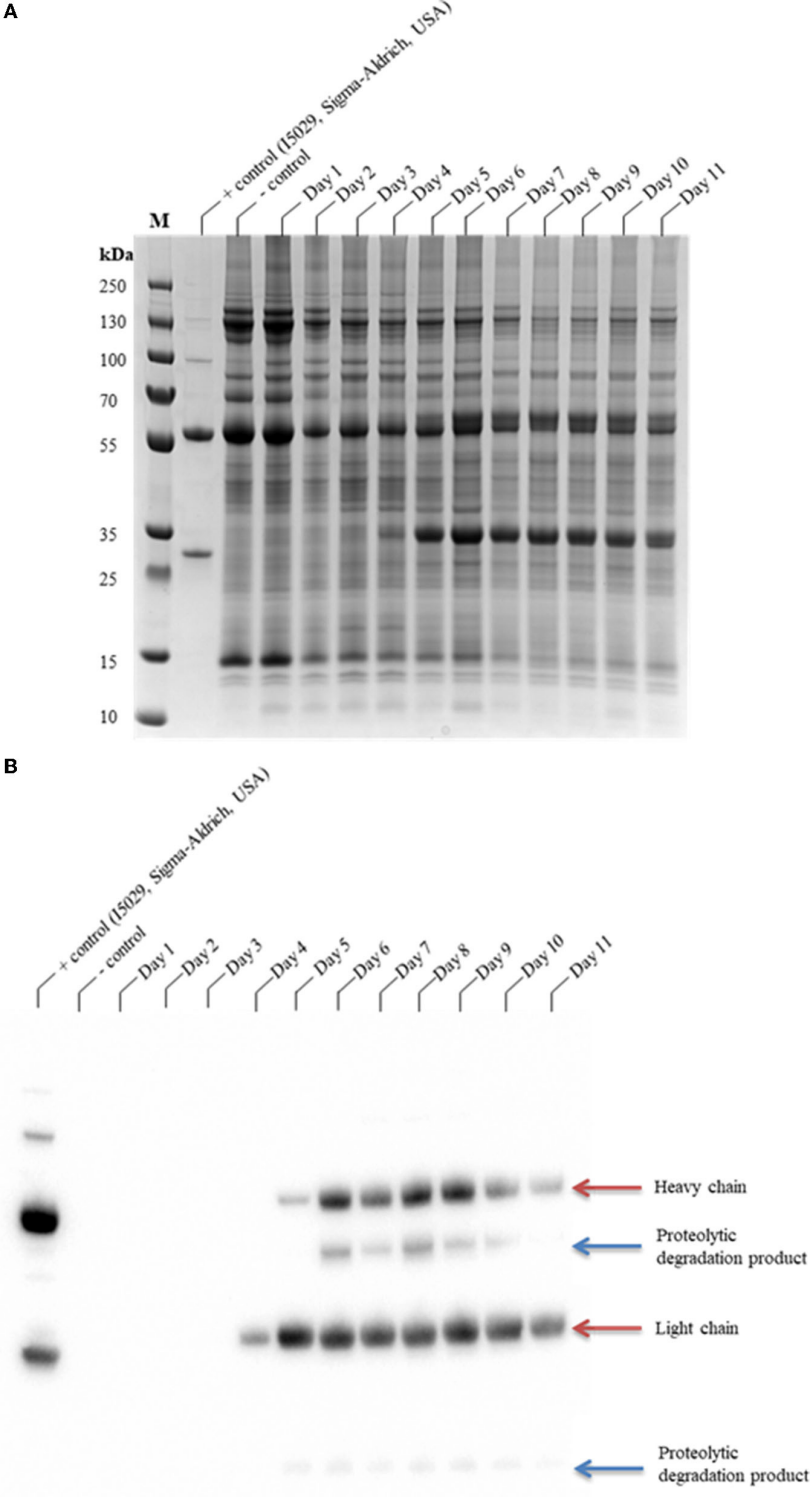
SDS-PAGE analysis of the production time profile of CAP256-VRC26.25 and its susceptibility to protease degradation. Samples were analyzed on a Bolt™ 4–12% Bis-Tris Plus gel **(A)** and thereafter assayed by Western blotting **(B)**. CAP256-VRC26.25 production in *N. benthamiana* (ΔXTFT) were assessed over 11 days, with 23.33 μg of protein assessed from each of the *N. benthamiana* (ΔXTFT) clarified extracts. M represents the molecular weight marker (PageRuler™ Plus Prestained Protein Ladder).

The glycoengineered *N. benthamiana* (ΔXTFT) (Strasser et al., 2008, 2009) host, which lacks glycan residues with core β1,2-xylose and α1,3-fucose moieties, was used for the transient expression of CAP256-VRC26.25. CAP256-VRC26.25 was coexpressed with hTPST1 (Loos et al., 2015; Singh et al., 2020) to enable tyrosine sulfation of the complementarity-determining region (CDR) H3. Expression levels were qualitatively assessed in clarified leaf extracts from 1 to 11 days post-infiltration (dpi) by SDS-PAGE (Figure 1A) and Western blot (Figure 1B). Under reducing conditions, detectable production of ~25 kDa band representing a LC by Western blot analysis was observed from day four, whereas production of ~50 kDa band representing an HC was observed from day five (Figure 1B). In addittion a ~10 kDa and ~40 kDa degradation species was observed from 5 to 10 dpi (Figure 1B) despite the addition of protease inhibitor cocktail to reduce *ex planta* degradation.

### Protease cleavage site analysis of the CAP256-VRC26.25 bNAbs

Putative cleavage sites for the C1, C13, and C14 protease families in the CAP256-VRC26.25 amino acid sequence were investigated *in silico* (Supplementary Table 1). Amino acids were designated by the single-letter code and cleavage sites were designated by arrows (↓), with the numbers appearing after amino acids referring to the position of potential cleavage. Highly abundant cleavage sites are highlighted in yellow, second highly abundant cleavage sites are highlighted in green, and highly scarce cleavage sites are highlighted in blue. The CAP256-VRC26.25 Ab consists of four subunits, two heavy and two light chains. *In silico* analysis revealed that the HC has 319 putative papain-like cleavage sites, 73 putative cathepsin L cleavage sites, and two putative legumain cleavage sites, whereas the LC has 105 putative papain-like cleavage sites, 27 putative cathepsin L cleavage sites, and two putative legumain cleavage sites. Liquid chromatography-tandem mass spectrometry (LC-MS/MS) confirmed that the intact IgG1 chains and degradation products, which were identified through Western blotting were indeed from CAP256-VRC26.25. Supplementary Figures 1–3 show the product ion MS/MS spectra of the three respective semi-tryptic peptides potentially due to cysteine protease action: DELTKNQVSLTCLV^397^↓KGFYPS, DELTKNQVSLTCLVKG^399^↓FYPS and DELTKNQVSLTCLVKGFY^401^↓PS, which lie within close proximity of one another in the Fc region of the CAP256-VRC26.25 HC.

### Transient reduction of *Nb*CysP6, *Nb*VPE-1a and *Nb*VPE-1b expression

Three strategies were taken: (i) targeting the *NbCysP6* alone, (ii) both *NbVPE-1a* and *NbVPE-1b*, and (iii) a combination of all three genes (*NbCysP6, NbVPE-1a* and *NbVPE-1b*) to reduce the proteolytic degradation of CAP256-VRC26.25. A nuclease assay approach, as described by Nekrasov et al. (2013), and qRT-PCR were used to determine successful transient genome editing. The T7 endonuclease cleavage assay was utilized to determine the success of genome editing, with the T7 endonuclease I recognizing and cleaving Holliday junctions. Successful mutagenesis of the *Nb*CysP6, *Nb*VPE-1a and *Nb*VPE-1b genes were carried out using Cas9, sgRNA:*Nb*CysP6 and sgRNA:*Nb*VPE1a/b in varying combinations. The success of the mutagenesis of *Nb*CysP6 can be observed (Supplementary Figure 4A), whereas (Supplementary Figure 4B) depicts the mutagenesis of *Nb*VPE-1a and *Nb*VPE-1b. Despite the concentration of DNA being normalized across all T7 endonuclease I reactions, varying degrees of cleavage efficiency were observed between all reactions. In general, we observed an increase of editing over time from three dpi to seven dpi for *Nb*CysP6, *Nb*VPE-1a, and *Nb*VPE-1b. The fragment size for both *Nb*VPE1a and *Nb*VPE1b was the same, making *Nb*VPE1a and *Nb*VPE1b fragment differentiation impossible.

**Figure 2:**
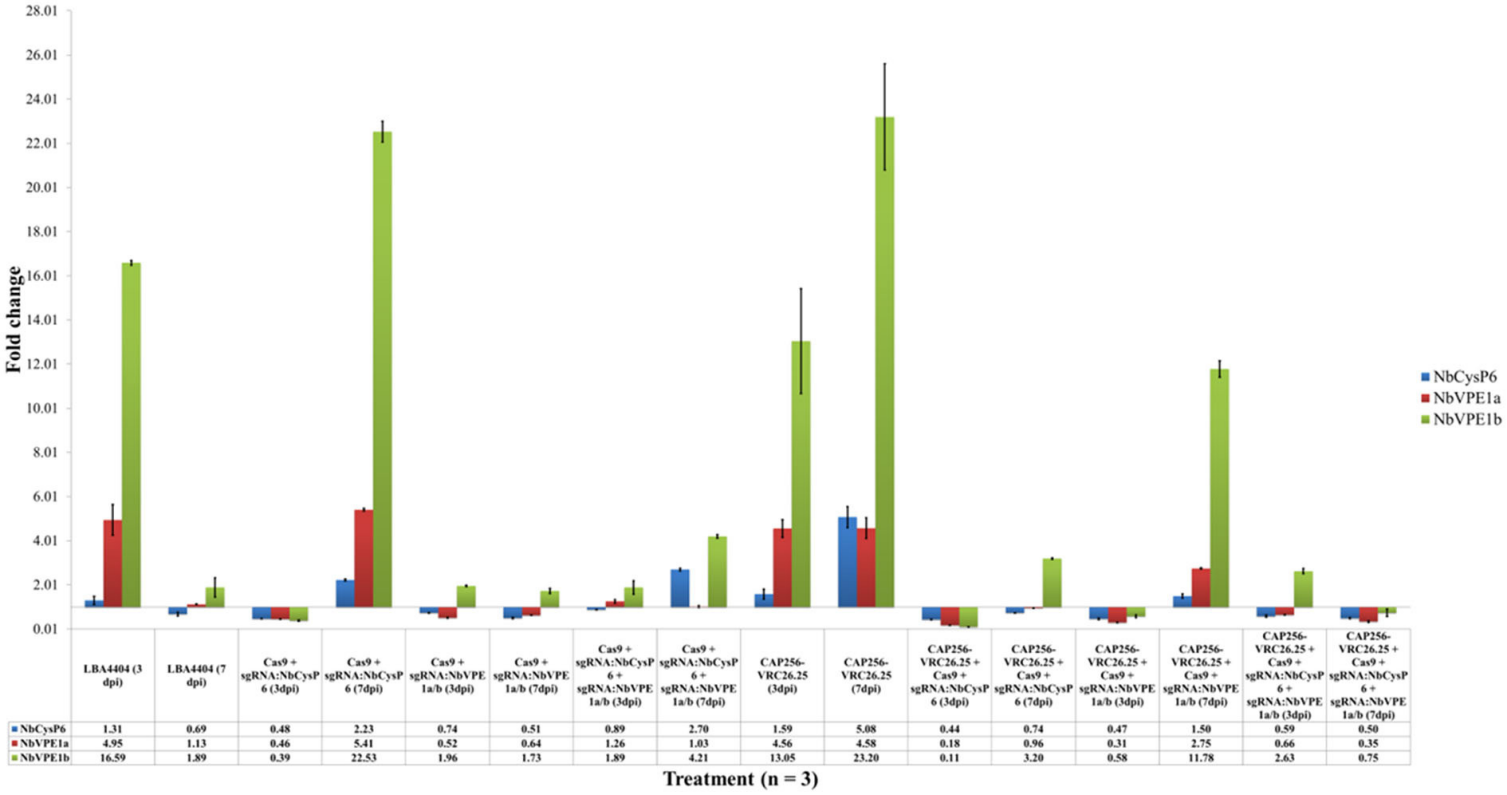
Fold change in the expression of *Nb*CysP6, *Nb*VPE1a and *Nb*VPE1b. Fluctuations in gene expression of *Nb*CysP6 (blue), *Nb*VPE1a (maroon), and *Nb*VPE1a (green) under all tested conditions were analyzed by RT-qPCR. Error bars indicate standard error of the mean (SEM) for three biological replicates. The table indicates the fold change in the expression of *Nb*CysP6, *Nb*VPE1a, and *Nb*VPE1b on three dpi and seven dpi. Experimental groups were normalized to reference genes and related to the uninfiltrated control set at one.

The disruption of the expression levels of CysP6 by means of RNAi was previously assessed by RT-qPCR (Duwadi et al., 2015). The gene expression levels of the investigated three cysteine proteases under uninfiltrated conditions were set to one. The upregulation of *Nb*CysP6, *Nb*VPE-1a, and *Nb*VPE-1b gene expression was measured at two key intervals, three and seven dpi, throughout the transient production period of CAP256-VRC26.25. Agroinfiltration of untransformed *A. tumefaciens* LBA4404 highly upregulated all three candidate protease genes, with *Nb*CysP6 being upregulated to a lesser extent than *Nb*VPE-1a and *Nb*VPE-1b (Figure 2). The expression of all three candidate protease genes showed a decrease from three to seven dpi, with only *Nb*CysP6 dropping below the gene expression levels under uninfiltrated conditions. Disruption of *Nb*CysP6 and/or *Nb*VPE-1a and *Nb*VPE-1b without CAP256-VRC26.25 production resulted in a similar change in the pattern of gene expression (Figure 2). CAP256-VRC26.25 expression without genome editing vectors resulted in high upregulation of all three cysteine proteases, in particular, *Nb*VPE-1b, with a fold change in gene expression from 13.05 to 23.20 from three dpi to seven dpi (Figure 2). Samples producing CAP256-VRC26.25, combined with the disruption of *Nb*CysP6, as well as samples targeting *Nb*VPE1a and *Nb*VPE1b, showed an initial decrease in gene expression of all three cysteine proteases at three dpi. These samples showed an increase in the gene expression levels of *Nb*VPE1b on seven dpi; with the samples producing CAP256-VRC26.25 combined with *Nb*VPE1a and *Nb*VPE1b disruption, showing an increase in *Nb*CysP6 and *Nb*VPE1a. Interestingly, when all three candidate protease genes were simultaneously disrupted with concomitant production of CAP256-VRC26.25, there was an overall decrease in gene expression levels of all three cysteine proteases to levels below that of the uninfiltrated control from three dpi to seven dpi (Figure 2).

### Transient reduction in proteolytic degradation of the CAP256-VRC26.25 bNAbs

**Figure 3:**
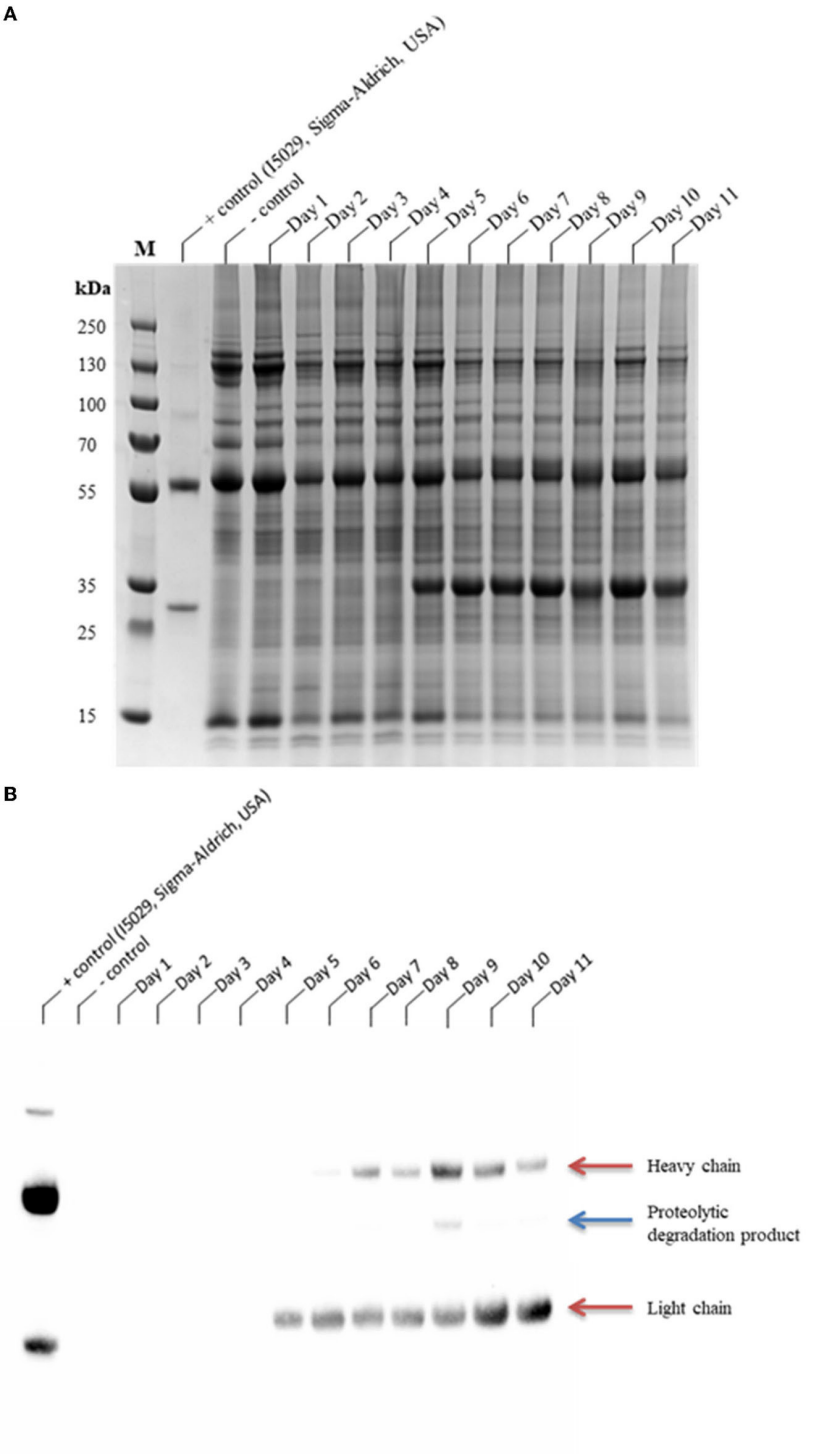
SDS-PAGE and Western blot analyses of the production time profile of the coexpression of CAP256-VRC26.25 with Cas9 and both sgRNA:*Nb*CysP6 and sgRNA:*Nb*VPE1a/b. Samples were analyzed on a Bolt™ 4–12% Bis-Tris Plus gel **(A)** and thereafter assayed by western blotting **(B)**. CAP256-VRC26.25 production with the coexpression of CRISPR/Cas9 and both sgRNA:*Nb*CysP6 and sgRNA:*Nb*VPE1a/b in *N. benthamiana* (ΔXTFT) was assessed over 11 days, with 23.33 μg of protein assessed from each of the *N. benthamiana* (ΔXTFT) clarified extracts. M represents the molecular weight marker (PageRuler™ Plus Prestained Protein Ladder).

**Figure 4:**
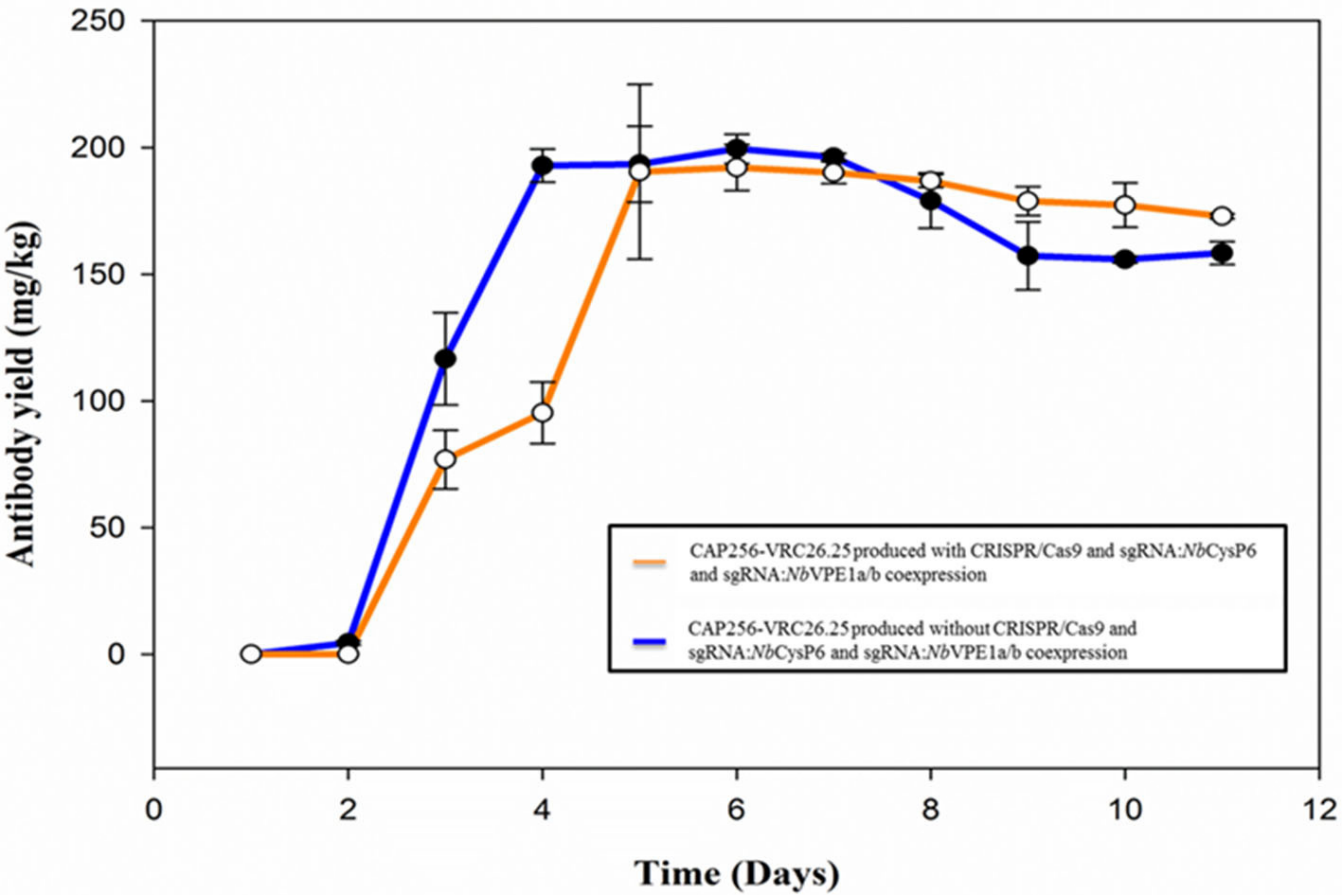
ELISA-determined production levels of CAP256-VRC26 mAbs produced *N. benthamiana* (ΔXTFT). The production of CAP256-VRC26.25 mAbs in *N. benthamiana* (ΔXTFT) was assessed over an 11-day period.

The coexpression of the individual sgRNAs targeting either *Nb*CysP6 or *Nb*VPE-1a and *Nb*VPE-1b showed a decrease in protease gene expression; however, qualitative analysis indicates that these strategies did not result in the reduction/elimination of the CAP256-VRC26.25 degradation species (Supplementary Figure 5). Interestingly, with simultaneous targeting of all three candidate protease genes and concomitant production of CAP256-VRC26.25, there was an overall decrease in gene expression levels of all three cysteine proteases to levels below that of the uninfiltrated control from three dpi to seven dpi (Figure 2) and a complete disappearance of the ~10 kDa and ~40 kDa degradation species (Supplementary Figure 5B). Qualitative and quantitative analysis of CAP256-VRC26.25 with the simultaneous targeting of all three genes was conducted from one to eleven dpi using SDS-PAGE (Figure 3A), western blot (Figure 3B), and ELISA (Figure 4). Under reducing conditions, detectable production of the LC by Western blot analysis was observed from day five, whereas detectable production of HC was observed from day six (Figure 3B). Some proteolytic degradation products are present on day nine, but this tapers off on day 10 and 11 (Figure 3B). Intact CAP256-VRC26.25 was detected through ELISA as early as two dpi, without the coexpression of genome editing vectors, however, with the coexpression of genome editing vectors, bNAbs was detected at three dpi. Without the coexpression of genome editing vectors, the concentration of produced bNAbs peaked at 6 dpi, with a concentration of 199.48 ± 5.75 mg/kg (Figure 4). With the coexpression of genome editing vectors, the concentration of produced intact bNAbs peaked at 6 dpi with a concentration of 192.09 ± 9.04 mg/kg (Figure 4).

### Effects of the disruption of candidate cysteine proteases on *in planta* cysteine protease activity

**Figure 5:**
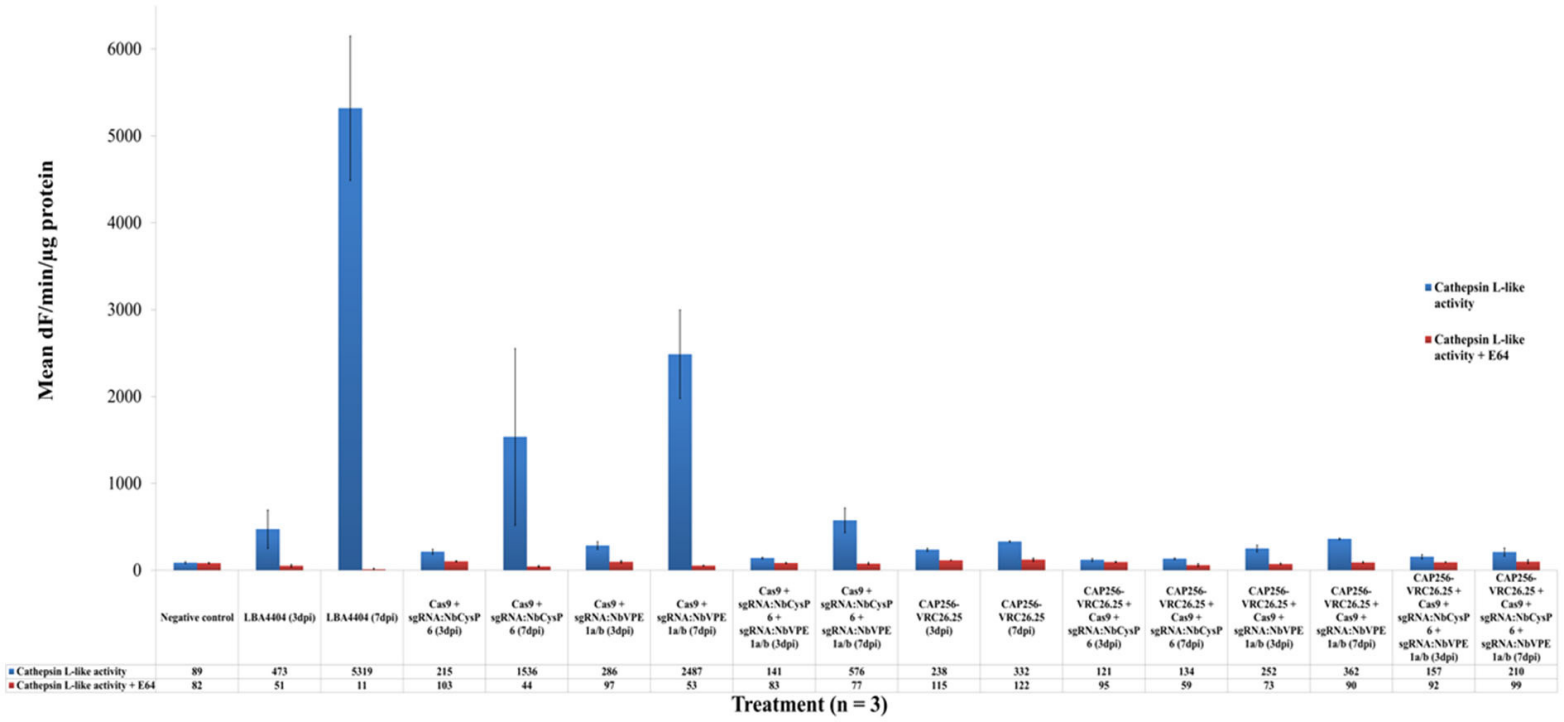
Cathepsin L-like protease activities of leaf samples with and without genome editing mediated disruption of proteases. Three dpi samples are in blue whereas seven dpi samples are in maroon. The y-axis represents the mean activities expressed as fluorescence units (dF) per min per μg protein. Mean activities of three biological replicates are shown within bars. Error bars indicate standard error of the mean (SEM).

**Figure 6:**
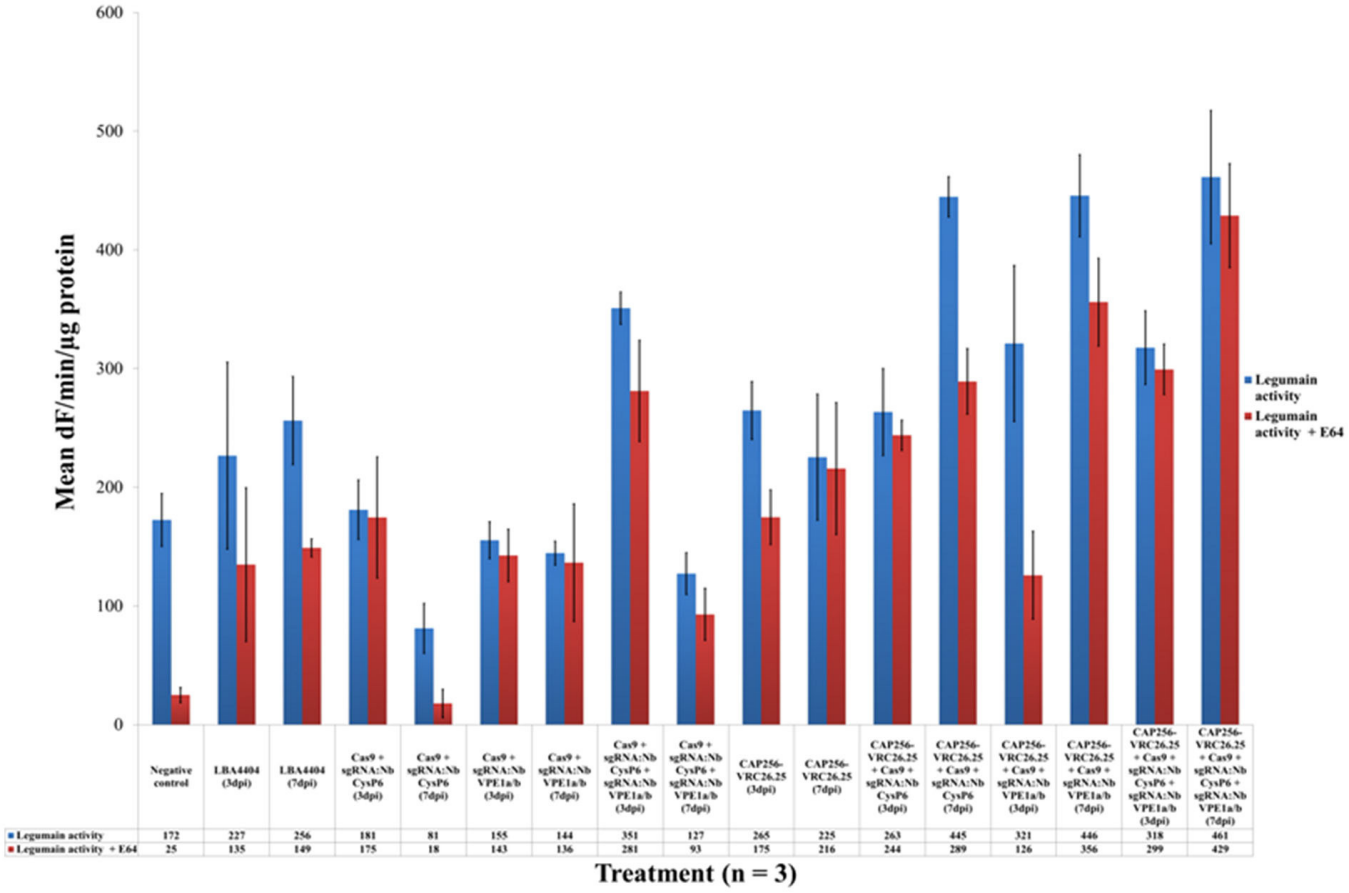
Legumain protease activities of leaf samples with and without genome editing mediated disruption of proteases. Three dpi samples are in blue whereas seven dpi samples are in maroon. The y-axis represents the mean activities expressed as fluorescence units (dF) per min per μg protein. Mean activities of three biological replicates are shown within bars. Error bars indicate standard error of the mean (SEM).

The influence of the change in gene expression of the three candidate genes on global cathepsin L-like (Figure 5) and legumain (Figure 6) activity of endogenous proteases within *N*. *benthamiana* (ΔXTFT) plants was also investigated. Statistically significant differences between samples, with or without CAP256-VRC26.25 production, combined without or with the disruption of *Nb*CysP6 and/or *Nb*VPE-1a and *Nb*VPE-1b, were determined by single-factor ANOVA with replication (*p* < 0.001) and *t*-tests (*p* < 0.05). Significant and highly significant differences were represented by ^*^ and ^**^, respectively, for cathepsin L-like (Supplementary Tables 6, 7) and legumain (Supplementary Tables 8, 9) protease activities. Samples without CAP256-VRC26.25 production, combined with the disruption of *Nb*CysP6 and/or *Nb*VPE-1a and *Nb*VPE-1b, showed an increase in cathepsin L-like activity from three to seven dpi (Figure 5). Untransformed *A. tumefaciens* LBA4404 had no influence on the legumain activity relative to the negative control, however, a huge spike in cathepsin L-like activity was observed from three to seven dpi. Disrupting *Nb*VPE-1a and *Nb*VPE-1b without CAP256-VRC26.25 production resultd in similar legumain activity to the uninfiltrated control. Interestingly, disrupting *Nb*CysP6 resulted in decreased legumain activity, suggestive of the involvement of *Nb*CysP6 in the regulation of proteases, which display legumain activity. CAP256-VRC26.25 production without editing vectors significantly increased cathepsin L-like activity relative to the uninfiltrated leaf sample, with an increase in cathepsin L-like activity from three to seven dpi.

CAP256-VRC26.25 production, combined with the disruption of *Nb*CysP6 on three and seven dpi, had significantly less cathepsin L-like activity compared to the three dpi samples of CAP256-VRC26.25 produced without genome editing vectors. CAP256-VRC26.25 production, combined with the disruption of *Nb*VPE1a and *Nb*VPE1b on seven dpi, had significantly higher cathepsin L-like activity. Cathepsin L-like activity was significantly lower for samples expressing CAP256-VRC26.25, combined with targeted disruption of *Nb*CysP6 on three and seven dpi, as well as samples producing CAP256-VRC26.25 combined with the disruption of all 3 candidate protease genes on three dpi relative to the seven-dpi sample of CAP256-VRC26.25 expression without genome editing vectors (Figure 5). Supplementary Tables 6, 7 show statistical differences between samples.

The legumain protease activity (Figure 6) of samples, with or without CAP256-VRC26.25 production, combined with or without the disruption of *Nb*CysP6 and/or *Nb*VPE-1a and *Nb*VPE-1b, was also measured. Disrupting either *Nb*CysP6 or all three candidate proteases without CAP256-VRC26.25 production showed a decrease in legumain activity from three to seven dpi, whereas disrupting *Nb*VPE1a and *Nb*VPE1b without CAP256-VRC26.25 production led to no notable change in legumain activity between three and seven dpi. Legumain activities on three and seven dpi of the leaf samples producing CAP256-VRC26.25 without genome editing vectors remain relatively unchanged. CAP256-VRC26.25 production in combination with genome editing vectors led to an increase in legumain activity (Figure 6), including samples in which reduction of *Nb*VPE-1a and *Nb*VPE-1b gene expression, was observed by RT-qPCR. There is an increase in legumain activity from three to seven dpi in CAP256-VRC26.25 samples. Supplementary Tables 8, 9 show statistical differences between samples.

### Effects of the disruption of candidate proteases on the host total soluble protein

**Figure 7:**
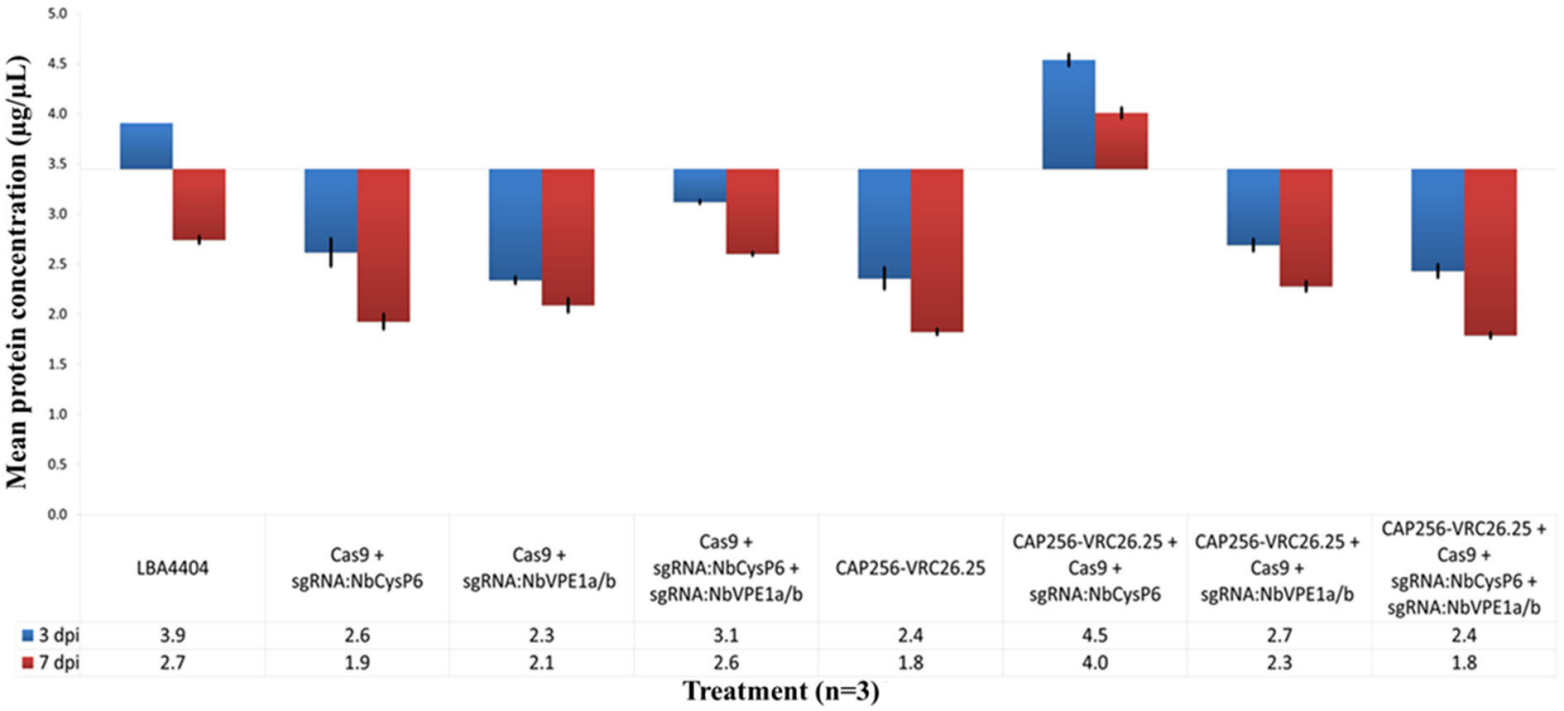
Effects of cysteine protease disruption on total soluble protein. Three dpi samples are in blue whereas seven dpi samples are in maroon. Error bars indicate standard error of the mean (SEM). The table indicates the mean TSP concentration on three dpi and seven dpi.

Changes in the levels of total soluble host proteins were used to determine the effects the disruption of the three-candidate protease had on host's cellular protein. The mean TSP concentrations in samples, with or without CAP256-VRC26.25 production, combined with or without the disruption of *Nb*CysP6 and/or *Nb*VPE-1a and *Nb*VPE-1b, were normalized to the uninfiltrated negative control set at 3.4 ± 0.068 μg/μL (Figure 7). Statistically significant differences between samples with and without the production of CAP256-VRC26.25 were determined by single-factor ANOVA with replication (*p* < 0.001) and *t-*tests (*p* < 0.05). Significant and highly significant differences were denoted by ^*^ and ^**^, respectively, (Supplementary Tables 10, 11). Disrupting *Nb*CysP6, both *Nb*VPE-1a and *Nb*VPE-1b, and a combination of *Nb*CysP6, *Nb*VPE-1a and *Nb*VPE-1b without CAP256-VRC26.25 production, resulted in a decrease in TSP from three to seven dpi, with all the samples presenting lower TSP levels than the uninfiltrated baseline. Producing CAP256-VRC26.25 (three and seven dpi) without genome-editing vectors led to significantly lower TSP than the negative control base line (Figure 7). Producing CAP256-VRC26.25, combined with either the disruption of both *Nb*VPE1a and *Nb*VPE1a or all 3 candidate protease genes, resulted in a decrease in TSP, with the lowest observed when all 3 candidate protease genes were targeted. Interestingly, the production of CAP256-VRC26.25 combined with the disruption of *Nb*CysP6 resulted in a significant increase in TSP three and seven dpi (Figure 7). This contrasts with *Nb*CysP6 disruption without CAP256-VRC26.25 production, which showed a reduction in host cell protein.

### Purification of CAP256-VRC26.25 produced in the presence and absence of genome editing vectors in *N. benthamiana* (ΔXTFT)

**Figure 8:**
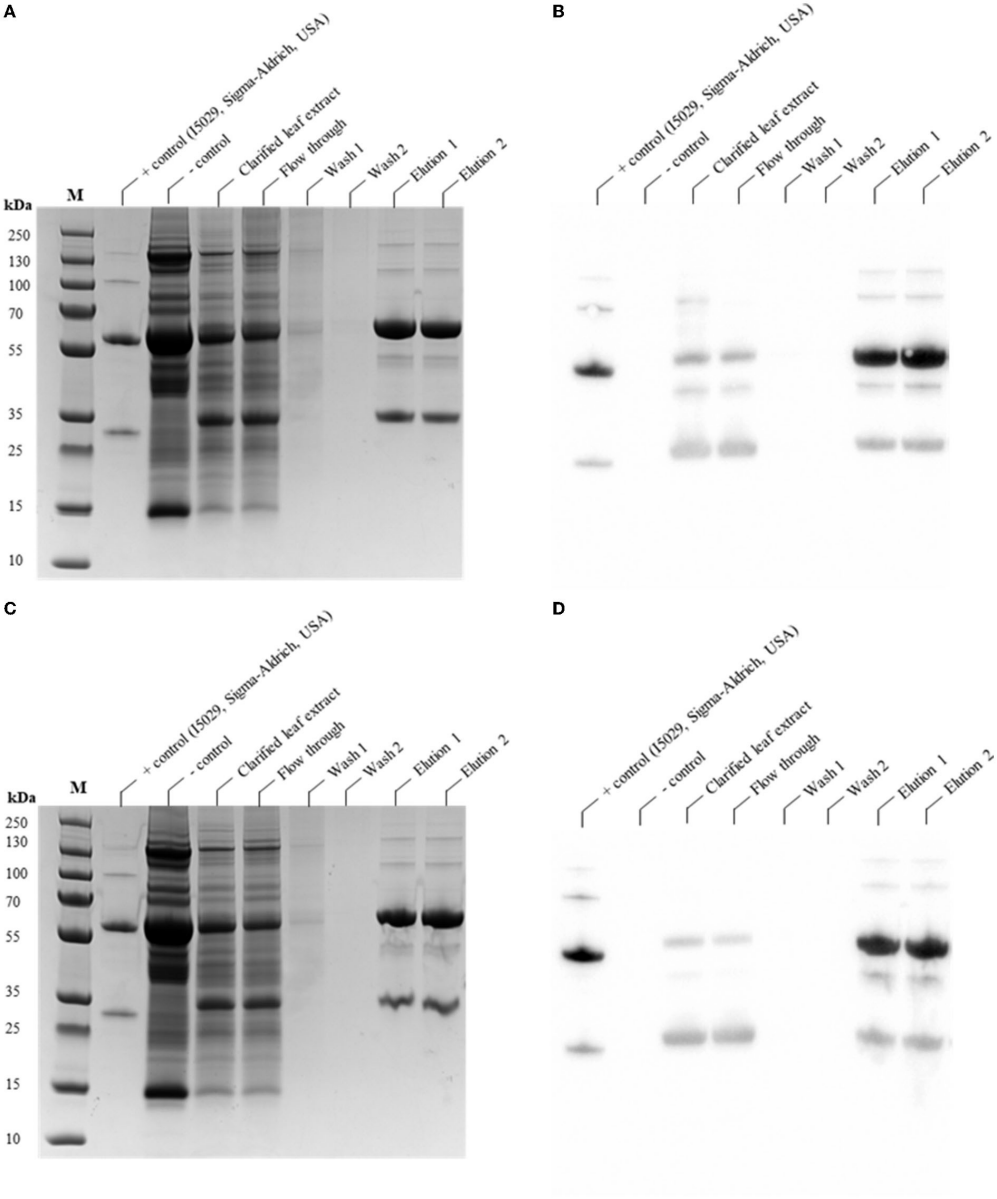
SDS-PAGE and Western Blot analysis of the purification of CAP256-VRC26 mAbs produced in *N. benthamiana* (ΔXTFT) with and without genome editing vectors. **(A,B)** SDS-PAGE and Western blot analysis of the purification of CAP256-VRC26.25 without genome editing vectors. **(C,D)** SDS-PAGE and Western blot analysis of the purification of CAP256-VRC26.25 produced with genome editing vectors. M represents the molecular weight marker (PageRuler™ Plus Prestained Protein Ladder).

Protein A based purification method is a common method for the purification of bNAbs, which results in >95% purity of the purified bNAb (Kim et al., 2018). The purification efficiency of CAP256-VRC26.25 produced without (Figures 8A,B) and with (Figures 8C,D) genome editing vectors was analyzed post-Protein A affinity purification by SDS-PAGE and Western blot analysis. The ~40 kDa proteolytic degradation product (Figure 8A) is shown to comprise two discrete products that has closely migrated. Both are likely the same polypeptide sequence; however, one species is post-translationally modified through glycosylation, while the other is not. Dectectable cleavage products were observed in the eluents of CAP256-VRC26.25 produced without genome editing vectors (Figures 8A,B). No detectable cleavage products by western blot were seen in the clarified extract and flow through of the CAP256-VRC26.25 produced with the simultaneous disruption of *Nb*CysP6 and *Nb*VPE1a/b (Figure 8D). Seven dpi was selected as a harvest point for the sample with disruption of *Nb*CysP6 and *Nb*VPE1a/b as no degradation product was observed (Figure 3B). There is, however, detectable amounts of protease degradation products that were found in the eluates (Figure 8D). This indicated that there was a low level of cleavage occurring and that the use of Protein A affinity purification has an enrichment effect on these proteolytic degradation products. Differences in expression efficiencies of recombinant protein has been previously observed in different leave types of *N. benthamiana* (Goulet et al., 2019). The observed low levels of cleavage can be as a result of a variation in the transient genome editing efficiency in small population of the harvested leaf material.

### Structural similarity between CAP256-VRC26.25 produced with and without protease disruption

**Figure 9:**
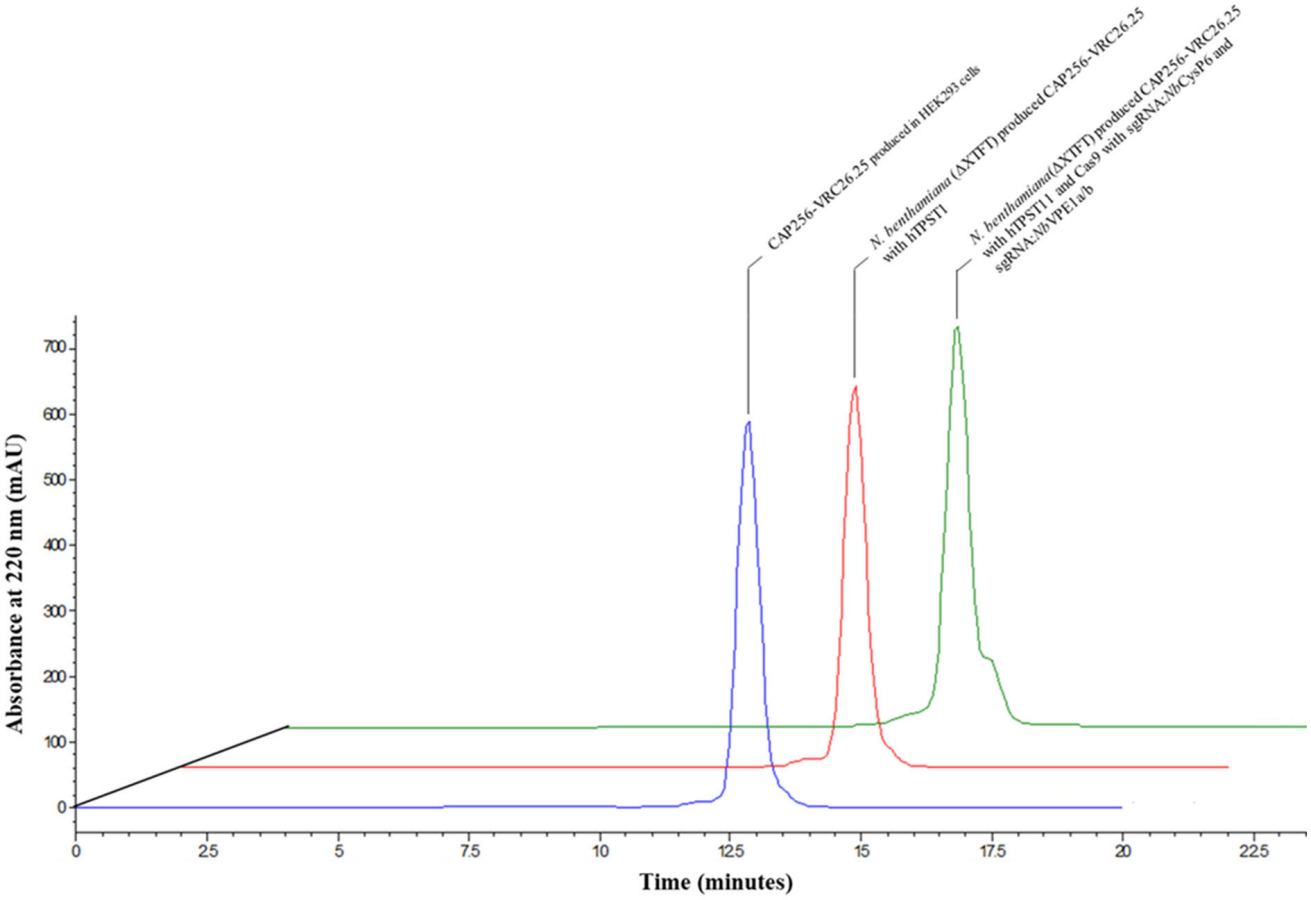
Overlaid chromatogram of the HPLC-SEC analysis of the CAP256-VRC26 mAbs. HPLC-SEC chromatogram of the CAP256-VRC26.25 mAbs analyzed under non-reducing conditions. Chromatogram of CAP256-VRC26.25 mAbs produced in HEK293 (blue). CAP256-VRC26.25 coexpressed with (green) and without (red) CRISPR/Cas9 and sgRNA:*Nb*CysP6 and sgRNA:*Nb*VPE1a/b in *N. benthamiana* (ΔXTFT) and CAP256-VRC26.25 produced in HEK293 (blue).

Size exclusion chromatography was used to characterize aggregates and levels of proteolytic degradation products in the antibody preparations. Comparative of the CAP256-VRC26.25 coexpressed with and without the simultaneous disruption of *Nb*CysP6 and *Nb*VPE1a/b in *N. benthamiana* (ΔXTFT) was done relative to the CAP256-VRC26.25 produced in HEK293. The overlaid views of SEC profiles for the Abs are shown in Figure 9. Each chromatogram was characterized by a peak at 13 min, representing single oligomeric state, (2LC, 2HC), of the intact CAP256-VRC26.25 antibody. There was no detectable cleaved fragment peak in all Ab samples, including the CAP256-VRC26.25 sample, produced in the absence of genome editing vectors; a sample known to contain proteolytic degradation fragments. A shoulder on the peak of the CAP256-VRC26.25 sample produced in the presence of genome editing vectors was noted. The identity of this shoulder is unclear as this shoulder is not observed in the CAP256-VRC26.25 sample, which was produced in the absence of genome editing vectors.

**Figure 10:**
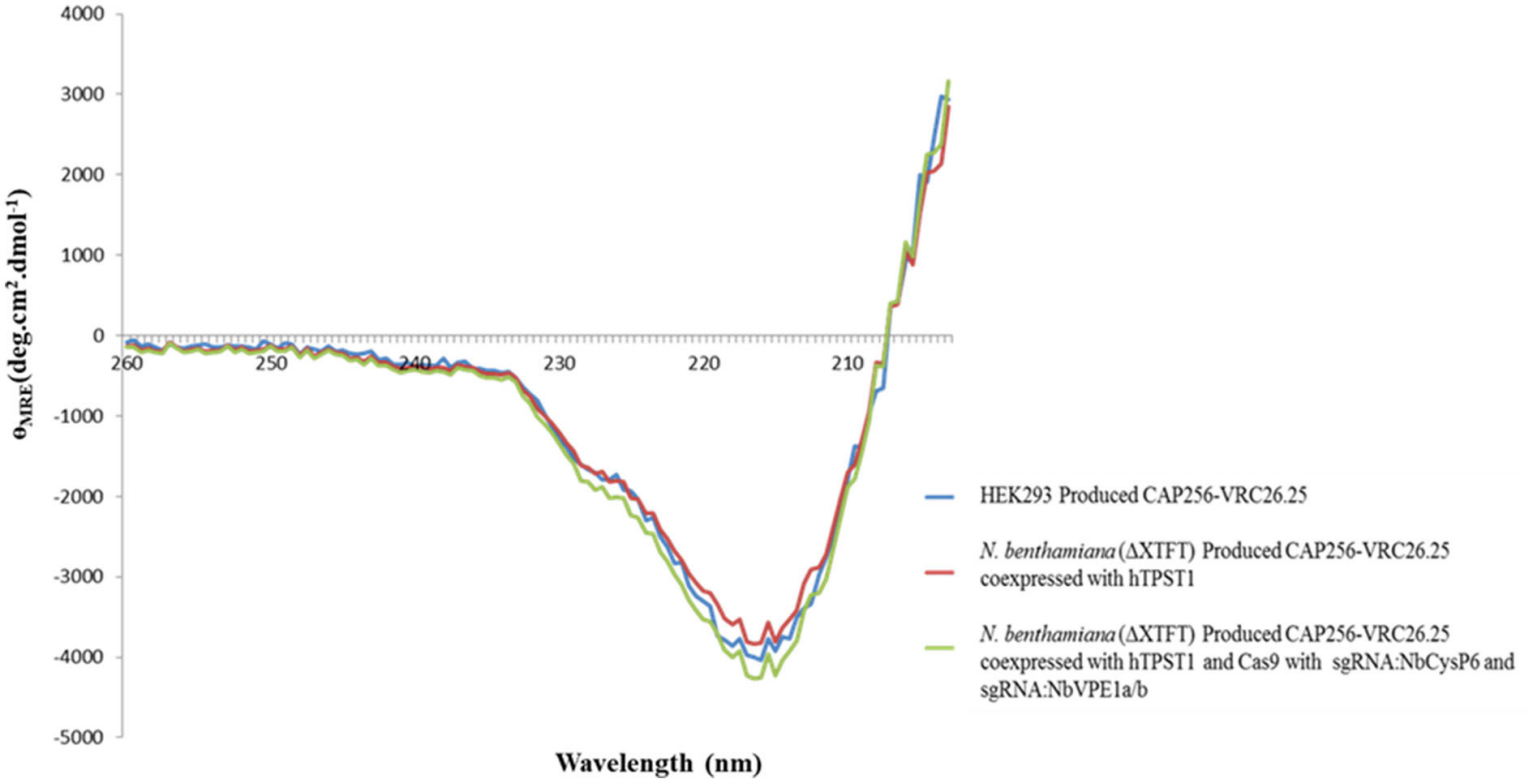
Far-UV CD spectra of CAP256-VRC26.25 mAbs. Far-UV CD spectra of CAP256-VRC26.25 produced with (maroon) and without (green) CRISPR/Cas9 and sgRNA:*Nb*CysP6 and sgRNA:*Nb*VPE1a/b coexpression in *N. benthamiana* (ΔXTFT) and of CAP256-VRC26.25 produced in HEK293 (blue).

**Figure 11:**
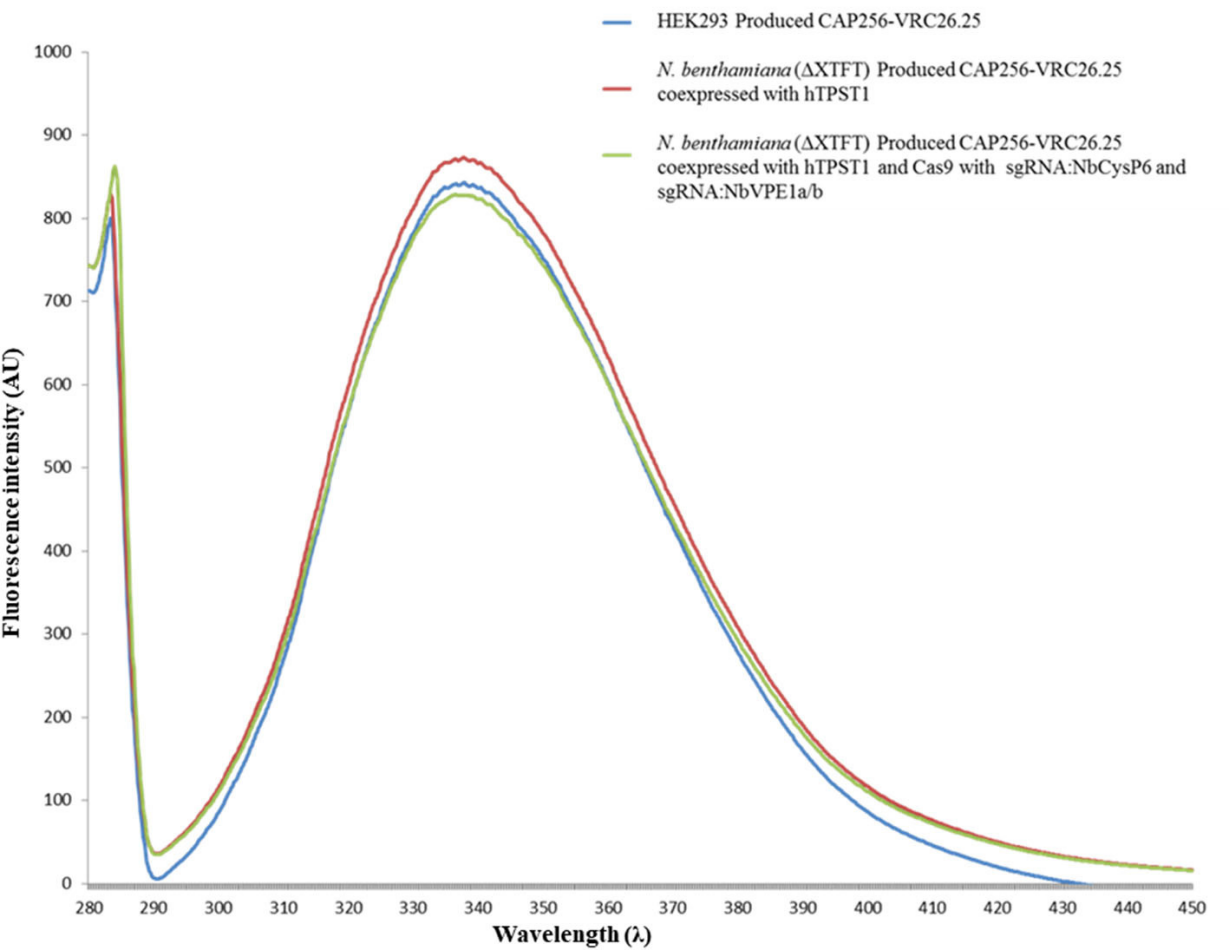
Fluorescence emission spectra of CAP256-VRC26.25 mAbs excited at 280 nm. Fluorescence emission spectra of CAP256-VRC26.25 produced with (maroon) and without (green) CRISPR/Cas9 and sgRNA:*Nb*CysP6 and sgRNA:*Nb*VPE1a/b coexpression in *N. benthamiana* (ΔXTFT) and of CAP256-VRC26.25 produced in HEK293 (blue).

**Figure 12:**
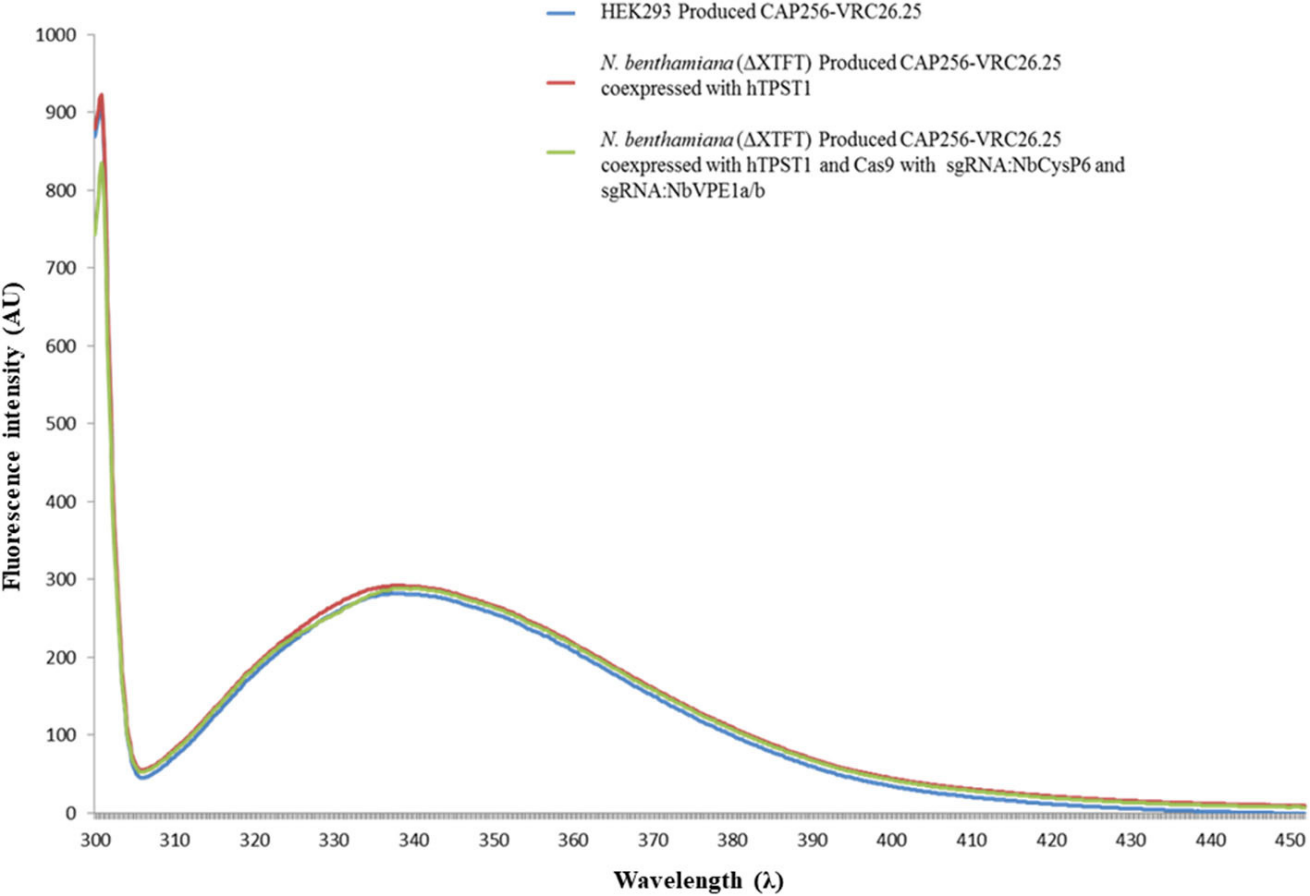
Fluorescence emission spectra of CAP256-VRC26.25 mAbs excited at 295 nm. Fluorescence emission spectra of CAP256-VRC26.25 produced with (maroon) and without (green) CRISPR/Cas9 and sgRNA:NbCysP6 and sgRNA:NbVPE1a/b coexpression in *N. benthamiana* (ΔXTFT) and of CAP256-VRC26.25 produced in HEK293 (blue).

Secondary and tertiary structural analysis of the CAP256-VRC26.25 produced with and without the disruption of *Nb*CysP6, *Nb*VPE1a, and *Nb*VPE1b in *N. benthamiana* (ΔXTFT) were assessed relative to the human embryonic kidney 293 (HEK293)-produced CAP256-VRC26.25 positive control. CAP256-VRC26.25 Abs' secondary structure was probed by circular dichroism (Figure 10). The CD spectra (Figure 10) displayed a characteristic minimum at 217 nm, characteristic of protein structures with dominant β-sheet content, such as the native Ab structures (Doi and Jirgensons, 1970). CAP256-VRC26.25 bNAbs produced with and without the disruption of *Nb*CysP6, *Nb*VPE1a and *Nb*VPE1b in *N. benthamiana* (ΔXTFT) had a similar secondary structural content to the HEK 293 produced Ab. Fluorescence spectroscopy was used to probe the folds of the CAP256-VRC26.25 Abs (Figures 11, 12). CAP256-VRC26.25 has 30 Trp and 54 Tyr residues, with all Trp and Tyr residues being distributed similarly throughout the Ab. The fluorescence spectral data of CAP256-VRC26.25 produced with and without the disruption of *Nb*CysP6, *Nb*VPE1a, and *Nb*VPE1b in *N. benthamiana* (ΔXTFT) relative to the HEK293 produced CAP256-VRC26.25 positive control depicted a λ_emmmax_ of 338 nm (Figures 11, 12).

### Equivalent *in vitro* HIV-1 neutralization efficacy between CAP256-VRC26.25 produced with and without protease disruption

CAP256-VRC26.25 is highly potent against HIV-1 subtypes A and C strains, with reduced potency against subtype B strains (Bhiman et al., 2015; Doria-Rose et al., 2015). CAP256-VRC26.25 produced in HEK293 and in *N. benthamiana* (ΔXTFT), with and without the disruption of *Nb*CysP6, *Nb*VPE-1a, and *Nb*VPE-1b, was tested against a multi-subtype panel of 15 pseudoviruses using the TZM-bl neutralization assay (Table 1). The IC_50_ values of HEK293-produced CAP256-VRC26.25 were similar to those obtained for *N. benthamiana* (ΔXTFT)-produced CAP256-VRC26.25, with and without the disruption of *Nb*CysP6, *Nb*VPE-1a, and *Nb*VPE-1b, neutralizing seven of the eight subtype C viruses and zero of the three subtype B viruses (Table 1). A similar pattern was observed for the neutralization of subtype A viruses, where CAP256-VRC26.25 produced in HEK293 and *N. benthamiana* (ΔXTFT) with and without the disruption of *Nb*CysP6, *Nb*VPE-1a and *Nb*VPE-1b neutralized three and four viruses, respectively. Importantly, the exceptional potency against some subtype C and A viruses was maintained for all CAP256-VRC26.25 samples, including that which was produced in *N. benthamiana* (ΔXTFT) without the disruption of all three proteases (Table 1).

**Table 1:** HIV-1 neutralizing activity of the CAP256-VRC26.25 bNAbs produced in *N. benthamiana* (ΔXTFT) without/with Cas9/sgRNA coexpression.

		**IC50 (**μ**g/mL)**		
		**CAP256-VRC26.25**		
**Subtype**	**Envelope**	**HEK293-produced mAbs**	***N. benthamiana* (ΔXTFT)-produced mAbs without Cas9/sgRNAs coexpression**	***N. benthamiana* (ΔXTFT)-produced mAbs with Cas9/sgRNAs coexpression**
C	Du422.01	0.11	0.10	0.20
	Du172.17	>50	>50	>50
	CAP210.E8	0.0012	0.0015	0.0014
	CAP45.G3	0.0013	0.0014	0.0013
	Du156.12	0.15	0.034	0.041
	ZM197.7	0.11	0.056	0.098
	ZM233.6	0.0044	0.0042	0.005
	CAP214.15	3.12	4.31	2.77
B	QH0692.42	>50	>50	>50
	TRO.11	>50	>50	>50
	6535	>50	>50	>50
A	Q461.e2	0.67	1.73	1.61
	Q168.a2	1.73	2.94	3.05
	Q23.17	0.0014	0.0016	0.0016
	Q842.d12	>50	>50	>50

## Discussion

*Nicotiana benthamiana* has been proven to be an adequate host for the production of efficacious V1V2-targeting HIV antibodies (Loos et al., 2015; Rosenberg et al., 2015; Singh et al., 2020). IgG1 antibodies have a characteristic of a ~ 50 kDa HC and a ~ 25 kDa LC two-band pattern under reducing conditions. Additional ~10 kDa and ~40 kDa bands were present in the *N. benthamiana* (ΔXTFT)-produced CAP256-VRC26.25 sample, similar to that which was observed in the *N. benthamiana* (ΔXTFT)-produced CAP256-VRC26.08 and CAP256-VRC26.09 samples (Singh et al., 2020). Fragment analysis confirmed four potential cleavage sites within proximity on each HC subunit of the CAP256-VRC26.25 Abs. Two putative papain cleavage sites and two putative cathepsin-L cleavage sites were identified, with no putative legumain cleavage sites predicted. Proteases from the PLCP and LLCPs have been shown to be upregulated in *N. benthamiana* in response to Agroinfiltration (Pillay et al., 2016; Grosse-Holz et al., 2018). PLCP and LLCPs have previously been implicated in the degradation of anti-HIV antibodies (Niemer et al., 2014; Duwadi et al., 2015; Paireder et al., 2017). More recently, two subtilisin-like serine proteases have more recently been identified for their roles in the degradation of recombinant proteins in *N. benthamiana* (Puchol Tarazona et al., 2021). In all instances, proteolytic cleavage is usually targeted to sterically exposed segments of the antibodies (Mandal et al., 2014; Niemer et al., 2014; Paireder et al., 2017; Puchol Tarazona et al., 2020). Given the sensitivity of many anti-HIV Abs to the above-mentioned LLCPs and PLCPs, it beckons the question as to whether CAP256-VRC26.25 is prone to degradation by these proteases. The vast majority of protease studies involve the knockout or downregulation of proteases through protease inhibitor coexpression, or pH control through the coexpression of proton channels (Girard et al., 2007; Pillay et al., 2012; Richau et al., 2012; Jutras et al., 2015). These methods have a broad inhibitory effect on all proteases and are not targeted to a specific protease of interest, and may result in unexpected changes in the physiology of the plant under defined growth conditions (Van der Vyver et al., 2003). The contributions of *Nb*CysP6, *Nb*VPE-1a, and *Nb*VPE-1b to the degradation of CAP256-VRC26.25, were elucidated in this study by the incorporation of a targeted CRISPR/Cas9-mediated genome-editing approach.

Protease gene expression was measured at two key intervals, three and seven dpi, throughout the transient production period of the CAP256-VRC26.25 bNAbs. Agroinfiltration of *N. benthamiana* (ΔXTFT) with untransformed *A. tumefaciens* LBA4404 had shown the upregulation of all three candidate cysteine proteases three dpi, with a decrease in gene expression of all three candidate cysteine proteases seven dpi. Apart from *A. tumefaciens* LBA4404, there are numerous commonly utilized *A. tumefaciens* strains, such as *A. tumefaciens* EHA105, *A. tumefaciens* GV3101, and *A. tumefaciens* GV3101::pMP90, which are used for the transient protein expression in *N. benthamiana*. It is uncertain as to whether each strain will induce the same protease gene expression profile as was observed for the untransformed *A. tumefaciens* LBA4404 sample. In this study, agroinfiltration of CAP256-VRC26.25 highly upregulated all the three candidate cysteine proteases on three dpi; these were further upregulated in seven dpi, in contrast to the untransformed *A. tumefaciens* LBA4404 sample. This difference in the gene expression profile can be accounted for by the vector system being utilized for the expression of the CAP256-VRC26.25 protein. This system relies on the co-infection and co-replication of two non-competing viral vectors, such as tobacco mosaic virus (TMV) and potato mosaic virus (PVX), which would further enhance the upregulation of these proteases (Giritch et al., 2006). The infection of tobacco leaves by TMV has been found to induce, and, ultimately, lead to programmed cell death (Hatsugai et al., 2004). A popular alternative is the pEAQ-HT expression system, which is based on the cowpea mosaic virus (CPMV) (Sainsbury et al., 2009; Peyret and Lomonossoff, 2013). This system has also been shown to enhanced the upregulation of these proteases, *Nb*CysP6, *Nb*VPE-1a, and *Nb*VPE-1b, as seen with the expression of the VP1 protein (Pillay et al., 2016). The full extent of the upregulation of these three cysteine proteases is unknown as sampling was done 24 h post-infiltration; as opposed to protease gene upregulation being monitored for the entire production period (Pillay et al., 2016). In our study, the coexpression of genome-editing vectors with CAP256-VRC26.25 showed successful reduction in gene expression of all three candidate cysteine proteases. The independent downregulation of *Nb*CysP6 or the downregulation of both *Nb*VPE-1a and *Nb*VPE-1b does not prevent/reduce the proteolytic degradation of CAP256-VRC26.25, despite a decrease in protease gene expression. Interestingly, it was only through simultaneous targeting of *Nb*CysP6, *Nb*VPE-1a, and *Nb*VPE-1b that we achieved a reduction in gene expression levels of all three cysteine proteases to lower the gene expression levels of these proteases under uninfiltrated conditions. This prominent reduction in protease gene expression also resulted in lower proteolytic degradation of CAP256-VRC26.25 bNAbs. ELISA-based quantification of the produced CAP256-VRC26.25 with and without *Nb*CysP6 and *Nb*VPE-1a/b disruption had suggested that the concentration of the oligomeric bNAb were essentially the same. The ELISA detection antibodies were the same as those used for detection in the western blots, which lacked the ability to differentiate between oligomeric bNAbs and degraded bNAbs fragments. This implied that the total concentration of the oligomeric bNAbs for the sample without *Nb*CysP6 and *Nb*VPE-1a/b disruption includes the quantification of degradation production.

Despite the absence of a putative legumain cleavage site in the CAP256-VRC26.25 Fc region, reduction of the proteolytic degradation products was only observed when all three candidate cysteine proteases were simultaneously downregulated, suggesting a synergistic function. Despite playing an important role in the regulation of physiological processes, the interaction between PLCPs and LLCPs and other proteases are hypothesized (Paulus and Van Der Hoorn, 2019). VPEs are autocatalytic proteases under acidic pH conditions and are known to activate PLCPs (Hiraiwa et al., 1999; Kinoshita et al., 1999; Müntz and Shutov, 2002).

The *Nb*CysP6 ortholog in *Arabidopsis*, RD21, recombinantly produced in insect cells did not self-mature itself, unless it was mixed with leaf extract (Yamada et al., 2001). RD21 is activated *via* a two-step mechanism involving other proteases that form part of a proteolytic cascade. It was suggested that VPEs plays a role in the activation of RD21 through this proteolytic cascade, however, formation of mature RD21 was shown to be possible in an *Arabidopsis* VPE quadrupole knockout mutant (Gu et al., 2012; Paulus and Van Der Hoorn, 2019). In contrast to RD21, *Nb*CysP6 is capable of self-activation. Recombinant *Nb*CysP6 was autocatalytic under acidic pH conditions, however, activation is not strictly limited to acidic pH conditions (Paireder et al., 2017). This suggests that the RD21-activation pathway may not be conserved between plant species (Paulus and Van Der Hoorn, 2019). The roles of PLCPs and LLCPs are synergistic as their upregulation is seen in many physiological processes, such as seed germination and hypersensitive response (Okamoto and Minamikawa, 1999; Zakharov et al., 2004). As seen with many proteases, our observations suggest that *Nb*CysP6, *Nb*VPE-1a, and *Nb*VPE-1b function synergistically in independent linear signaling cascades involved in various physiological processes (van der Hoorn and Jones, 2004).

Proteolytic activity, cathepsin L-like and legumain activity, and protease gene expression were analyzed to determine if there was a correlation among *Nb*CysP6, *Nb*VPE-1a, and *Nb*VPE-1b expressions and activities in the degradation of CAP256-VRC26.25. When infiltrated by untransformed *A. tumefaciens* LBA4404, there was no increase in legumain activity relative to the negative control. There was, however, a huge spike in cathepsin L-like activity from three to seven dpi. It is unlikely that *Nb*CysP6 is solely responsible for this spike in activity as RT-qPCR shows a decrease in the gene expression of *Nb*CysP6 over time. The MEROPS database currently lists a total of 516 putative peptidases and 97 non-peptidase homologs (Rawlings et al., 2018); amongst these, other listed cysteine proteases could have contributed to this overall increase in activity. Apart from *Nb*CysP6, other proteases from the C1 family are upregulated in response to infiltration with untransformed *A. tumefaciens* LBA4404, of which 41 members form part of the C1 family (Pillay et al., 2016; Grosse-Holz et al., 2018). The downregulation of *Nb*VPE-1a and *Nb*VPE-1b in the control samples resulted in similar legumain activities to the negative control. Interestingly, the downregulation of *Nb*CysP6 results in a decrease in legumain activity, suggesting the possibility of *Nb*CysP6 involvement in the regulation of proteases, which display legumain activity. Infiltration of CAP256-VRC26.25 without genome-editing vectors did not influence legumain activity. There was an increase in cathepsin L-like activity from three to seven dpi, however, this was as high as those observed for plants infiltrated with *A. tumefaciens* LBA4404 alone. Samples where CAP256-VRC26.25 was coexpressed with genome-editing vectors showed an increase in legumain activity, including samples, in which reduction of *Nb*VPE-1a and *Nb*VPE-1b gene expression was observed by RT-qPCR. There is an increase in legumain activity from three to seven dpi in CAP256-VRC26.25 infiltrated samples, the upregulation of metacaspase-1 as previously seen with the agroinfiltration of VP1 could account for this observation (Pillay et al., 2016). Despite the knowledge of the protease suite in *N. benthamiana* being quite comprehensive, deducing a definitive correlation from the proteolytic activity data should be done with caution because proteases function in cascades, which may cause an upregulation of other proteases to compensate for the disruption of a particular protease (Grosse-Holz et al., 2018; Kourelis et al., 2019).

Changes in total soluble host protein levels were used to determine the effects of downregulating *Nb*CysP6, *Nb*VPE-1a, and *Nb*VPE-1b on host cellular protein. Agroinfiltration triggers senescence, of which the first organelle to be disorganized is the chloroplast, which holds 75% of the leaf nitrogen, in the form of Rubisco. Degradation of the chloroplastic protein results in reduced photosynthetic capacity of the leaf (Hörtensteiner and Feller, 2002). The agroinfiltration of genome-editing vectors in the absence of the CAP256-VRC26.25 constructs had shown a decrease in host cell protein, with the extent of the decrease in host cell protein increasing seven dpi. Three samples deviated from this pattern, the agroinfiltration of untransformed *A. tumefaciens* LBA4404 on three dpi, and the downregulation of *Nb*CysP6 with CAP256-VRC26.25 expression on three and seven dpi. The infiltration of untransformed *A. tumefaciens* LBA4404 had initially showed an increase in host cell protein three dpi, however, following this there was a decrease in host cell protein. The initial stages of senescence involve the upregulation of PR protein, which was why an increase in host cell protein was observed in contrast to the CAP256-VRC26.25 samples in the absence of genome editing vectors. This observation was possibly due to the *A. tumefaciens* LBA4404 not being transformed with deconstructed viral expression vectors, which may induce the functionality of these PR proteins in a manner that was observed with the CAP256-VRC26.25 samples in the absence of genome-editing vectors, in which a decrease in host cell protein three dpi was observed. A decrease in host cell protein from three dpi to seven dpi was observed in the *A. tumefaciens* LBA4404 samples, indicating that in the absence of deconstructed viral vectors, the function of these PR proteins occurs at a slower rate than those observed with samples containing deconstructed viral expression vectors. Most interestingly, the downregulation of *Nb*CysP6 when CAP256-VRC26.25 was expressed resulted in a concomitant increase in host cell protein on three and seven dpi. This contrasts with the *Nb*CysP6 downregulated sample without coexpressed bNAb, which showed a reduction in host cell protein. Interestingly, despite the combined downregulation of *Nb*CysP6, *Nb*VPE-1a, and *Nb*VPE-1b preventing the degradation of CAP256-VRC26.25, this sample showed a reduction in host cell protein, suggesting that the protein responsible for Rubisco degradation retained full functionality.

Overall, the technique of agroinfiltration induces the hypersensitive response, which upregulates a suite of proteases in addition to *Nb*CysP6, *Nb*VPE-1a, and *Nb*VPE-1b, which are involved PCD. This might explain the overall decrease in host cellular proteins in most samples prepared in this study. Rubisco can be hydrolyzed in intact chloroplasts or in chloroplast lysates, and it has been suggested the initial stages of senescence degradation of the plastidial proteins occurs inside the organelle (Feller et al., 2008; Kato and Sakamoto, 2010). The chloroplastic environment is rich in serine and metalloproteases, suggesting these to be the prime candidates for initial Rubisco degradation (van der Hoorn, 2008). An upregulation of metalloproteases from two families (M38 and M67) were previously seen in response to agroinfiltration, thereby degrading host cell protein (Pillay et al., 2016). The involvement of cysteine proteases in Rubisco degradation was investigated with the use of E64, a commonly used cysteine protease inhibitor. Rubisco degradation was reduced in the presence of this protease inhibitor (Carrión et al., 2013). *Arabidopsis* has three different genes, which encode the cysteine protease Cathepsin B, which are upregulated in developmental leaf senescence. Mutation of all three Cathepsin B genes delayed senescence, when monitored by observed loss of chlorophyll. Cathepsin B may act upstream of other proteases involved in senescence, of which the expression is reduced in the triple mutant Cathepsin B plant. This places Cathepsins in a pathway regulating senescence; however, the targets of Cathepsin B are unknown (Pružinská et al., 2017).

Characterization and quantification of the cleaved states and aggregates is of particular importance for biopharmaceuticals as these species could potentially affect the efficacy of the bNAbs. A single oligomeric state was detected, with no observed peak, which could be indicative of the presence of proteolytic degradation products. This is suggestive, that the proteolytic degradation fragment is still associated with the Ab structure. Apart from the disulfide bridging between the LCs and HCs, strong non-covalent associations exist between the two paired HCs in the CH3 regions (Dall'Acqua et al., 1998; van der Neut Kolfschoten et al., 2007). These strong non-covalent forces are responsible for the non-dissociating cleaved fragment in this previous study (Brezski et al., 2009). Non-denaturing SEC studies on a intact IgG1s and a single-cleaved IgG1s (IgG which is cleaved at a particular site on one HC chain) were indistinguishable (Brezski et al., 2009). This is consistent with observations made under non-reducing conditions of the *N. benthamiana* (ΔXTFT)-produced CAP256-VRC26.08 and CAP256-VRC26.09, in which a single intact band was observed (Singh et al., 2020). However, it is unclear in this current study as to whether CAP256-VRC26.25 is cleaved on a single HC, or both, contributing to this observation or whether this is due to the low abundance of the cleaved fragment. The structural similarity between the CAP256-VRC26.25 bNAbs produced in HEK293, and in *N. benthamiana* (ΔXTFT) with and without the simultaneous disruption of *Nb*CysP6 and *Nb*VPE-1a/b. Despite the presence of protein degradation products in *N. benthamiana* (ΔXTFT) CAP256-VRC26.25 samples produced without protease disruption, there were no detectable secondary, tertiary, and quaternary structural differences when compared to the HEK293 produced bNAbs and with simultaneous disruption of *Nb*CysP6 and *Nb*VPE1a/b.

As seen with CAP256-VRC26.08 and CAP256-VRC26.09, the CAP256-VRC26.25 bNAbs produced in *N. benthamiana* (ΔXTFT) without protease disruption, had the same *in vitro* neutralization potency as the HEK293-produced bNAbs. CAP256-VRC26.25 produced in HEK293 and *N. benthamiana* (ΔXTFT) with and without the simultaneous disruption of *Nb*CysP6 and *Nb*VPE-1a/b all displayed high potency against HIV-1 subtype A and C strains, with reduced potency against subtype B strains, consistent with previous work (Bhiman et al., 2015; Doria-Rose et al., 2015). The Fab region of the CAP256 antibodies recognizes antigens and activates (or suppress) immune cells through interactions with and between the Fc region and the cell bearing Fc receptors (FcR). The *in vitro* TZM-bl assay used in this study assays the neutralization of the Fab region of the CAP256 antibodies to the V1V2 region of the HIV-1 gp120 envelope glycoprotein. The effects of the cleaved Fc region of the CAP256-VRC26.25 bNAbs produced in *N. benthamiana* (ΔXTFT) without genome-editing vectors were not detectable by the TZM-bl assay. Binding of the Fc region to the FcRs activates antibody-dependent cellular cytotoxicity (ADCC), antibody-dependent cellular phagocytosis (ADCP), or complement- dependent cytotoxicity (CDC). The serum half-life of IgGs is mediated by the pH-dependent binding to the neonatal Fc receptor (FcRn) (Datta-Mannan et al., 2007). Residues, which are responsible for FcRn binding are located between the CH2 and CH3 regions of the HC (Kim et al., 1999; Roopenian and Akilesh, 2007). One of these residues is N297, the glycosylation which influences both the structure and function of the IgG. Potential cleavage sites were identified between amino acid positions 397 and 401, indicative of the likely retention of glycosylation even in proteolytically degraded bNAbs. From this study it is uncertain as to whether a single or both CAP256-VRC26.25 HCs are cleaved. It was previously observed that IgG1s with single cleavage in the hinge region maintain affinity to the FcRn; however, they showed a loss of affinity to the Fcγ family of receptors (Brezski et al., 2009; Brezski and Jordan, 2010). The effects of truncated bNAbs on *in vivo* potency and humoral immunity are yet to be determined.

## Conclusion

From this study and other studies, endogenous proteolytic degradation is a bottleneck in the production of antibodies in planta. This study has shown that this bottleneck can be reduced/avoided through the transient's downregulation of proteases like *Nb*CysP6, *Nb*VPE-1a, and *Nb*VPE-1b, thus, making it possible to produce intact highly potent anti-HIV and other Abs. Though the functional activity of these bNAbs has not been demonstrated *in vivo*, it is still crucial to consider proper downstream processing to improve the overall quality of the produced protein to avoid any aberrant immune responses, which may arise from the presence of these truncated antibodies and proteolytic fragments. The generation of a stable mutant with the *Nb*CysP6, *Nb*VPE-1a, and *Nb*VPE-1b gene knocked out will eliminate the low levels of cleavage observed through Protein A enrichment during the purification process. For *in planta-*produced Abs to gain further wide-spread acceptance and adoption as an industry alternative to produce biopharmaceuticals, the findings of this study may be key considerations for the biopharma sector.

## Data availability statement

The raw data supporting the conclusions of this article will be made available by the authors, without undue reservation.

## Author contributions

RC, AS, PP, and TT conceived the study. RC, PP, TT, and JV supervised the study. AS and PP conducted all antibody expressions and genome-editing experimentations. KA provided technical input on neutralization assays, with neutralization assays being conducted by KM. LM and HS designed and optimized hTPST1 construct. HS provided the *N. benthamiana* (ΔXTFT) seeds used in this study. AS, PP, PN, and KA performed the experiments. AS, PP, PN, KA, and TT analyzed the data. AS, PP, and TT designed the experiments and wrote the manuscript with input from LyM, JB, LM, and HS. All authors contributed to the article and approved the submitted version.

## Funding

This study was funded by the Department of Science and Innovation (DSI), South African Medical Research Council–Strategic Health Innovation Partnership (SAMRC SHIP), National Research Foundation (NRF), Council for Scientific and Industrial Research (CSIR), and the CSIR: Young Researcher Establishment Fund (Grant No.: YREF 2022 13).

## Conflict of interest

The authors declare that the research was conducted in the absence of any commercial or financial relationships that could be construed as a potential conflict of interest.

## Publisher's note

All claims expressed in this article are solely those of the authors and do not necessarily represent those of their affiliated organizations, or those of the publisher, the editors and the reviewers. Any product that may be evaluated in this article, or claim that may be made by its manufacturer, is not guaranteed or endorsed by the publisher.
