# Corrigendum: Transient proteolysis reduction of *Nicotiana benthamiana*-produced CAP256 broadly neutralizing antibodies using CRISPR/Cas9

**DOI:** 10.3389/fpls.2025.1568756

**Published:** 2025-03-19

**Authors:** Advaita Acarya Singh, Priyen Pillay, Previn Naicker, Kabamba Alexandre, Kanyane Malatji, Lukas Mach, Herta Steinkellner, Juan Vorster, Rachel Chikwamba, Tsepo L. Tsekoa

**Affiliations:** ^1^ Future Production: Chemicals Cluster, Council for Scientific and Industrial Research, Pretoria, South Africa; ^2^ Department of Plant and Soil Sciences, University of Pretoria, Pretoria, South Africa; ^3^ NextGen Health Cluster, Council for Scientific and Industrial Research, Pretoria, South Africa; ^4^ Department of Applied Genetics and Cell Biology, University of Natural Resources and Life Sciences, Vienna, Austria

**Keywords:** plant biotechnology, CRISPR/Cas9, genome editing, *Nicotiana benthamiana*, proteases, immunoglobulin G, human immunodeficiency virus

In the published article, there was an error. In the original article, we did not have the Addgene repository numbers and hyperlinks available so we could not refer to them and so that information was omitted from the original article.

A correction has been made to **Materials and methods**, *The sgRNA cloning*. This sentence previously stated:

“Vectors were transformed into *E. coli* DH10β and sequenced by Inqaba Biotechnical Industries (Pty) Ltd (ZA) using sequencing primers listed in Supplementary Table 2.”

The corrected sentence appears below:

“Vectors were transformed into *E. coli* DH10β and sequenced by Inqaba Biotechnical Industries (Pty) Ltd (ZA) using sequencing primers listed in Supplementary Table 2. Plasmids were deposited in Addgene under the following names and IDs:pICH86966::AtU6p::sgRNA: NbCysP6 (223217) and pICH86966::AtU6p::sgRNA: NbVPE1a/b (223218).”

The authors apologize for this error and state that this does not change the scientific conclusions of the article in any way. The original article has been updated.

