# Supplementary material for: Transient proteolysis reduction of Nicotiana benthamiana-produced CAP256 broadly neutralizing antibodies using CRISPR/Cas9
Source: Front Plant Sci. 2022 Aug 18;13:953654. doi: 10.3389/fpls.2022.953654 (PMC9433777; doi:10.3389/fpls.2022.953654)
Supplement: Supplementary file 1 [file Data_Sheet_1.zip › Supplementary Material.docx]

Transient Proteolysis Reduction of *Nicotiana benthamiana*-Produced CAP256 Broadly Neutralising Antibodies using CRISPR/Cas9

Advaita Acarya Singh^1,4^, Priyen Pillay^1^, Previn Naicker^2^, Kabamba Alexandre^2^, Kanyane Malatji^2^, Lukas Mach^3^, Herta Steinkellner^3^, Juan Vorster^4^, Rachel Chikwamba^1^ and Tsepo L. Tsekoa^1^*

Supplementary Material

## Supplementary Figures

**Supplementary figure legend**

**Supplementary figure S1| Representative product ion MS/MS spectrum of the DELTKNQVSLTCLV semi-tryptic peptides of the cleaved CAP256-VRC26.25 HC.** m/z of each precursor ion and their monoisotopic masses are represented in the fragmentation spectra above. Cleavage site analysis was done with a confidence of 95%.

**Supplementary figure S2| Representative product ion MS/MS spectrum of the DELTKNQVSLTCLVKG semi-tryptic peptides of the cleaved CAP256-VRC26.25 HC.** m/z of each precursor ion and their monoisotopic masses are represented in the fragmentation spectra above. Cleavage site analysis was done with a confidence of 95%.

**Supplementary figure S3| Representative product ion MS/MS spectrum of the DELTKNQVSLTCLVKGFY semi-tryptic peptides of the cleaved CAP256-VRC26.25 HC.** m/z of each precursor ion and their monoisotopic masses are represented in the fragmentation spectra above. Cleavage site analysis was done with a confidence of 95%.

**Supplementary figure S4|** TBE-acrylamide gel qualitatively analysing the success of CRISPR/Cas9 mediated genome editing. (A) Analysis of T7 Endonuclease I assay to determine successful editing of the *Nb*CysP6 gene. (B) Analysis of T7 Endonuclease I assay to determine successful editing of the *Nb*VPE1a and *Nb*VPE1b gene. M represents the DNA ladder.

**Supplementary figure S5| SDS-PAGE and Western blot analysis of the coexpression of CAP256-VRC26.25 with Cas9 and sgRNAs in *N. benthamiana* (ΔXTFT).** (A) and (B) SDS-PAGE and Western blot analysis of the coexpression of CAP256-VRC26.25 with Cas9 and sgRNA in *N. benthamiana* (ΔXTFT). 7.63 µg of protein assessed from each of the *N. benthamiana* (ΔXTFT) clarified extracts. M represents the molecular weight marker (PageRuler™ Plus Prestained Protein Ladder).

## Supplementary Tables

**Supplementary table S1| *In silico* proteolytic cleavage site prediction for the heavy and light chain of CAP256-VRC26.25 using primarily the P1 and P2 substrate positions within amino acid sequences.**

| **Protease** | **Light chain** | **Heavy chain** | **Cleavage** | **Preferential cleavage** |
| --- | --- | --- | --- | --- |
| ***Cysteine protease*** |  |  |  |  |
| **Papain (C^25^, H^159^, N^175^)** | DR^62^, QR^40^ | VR^38^, LR^19, 87, 101^, FR^28^, PR^320, 372^, SR^72,^ ^125,^ ^283,^ ^383,^ ^444^ | AR↓, VR↓, LR↓, IR↓, FR↓, WR↓, YR↓, TR↓, MR↓, PR↓, DR↓, SR↓, ER↓, QR↓ | (A, V, L, I, F, W, Y – hydrophobic amino acids in P2 position)  (↓ no Val after cleavage sites) |
|  | VK^61^, WK^154^, PK^115^, DK^54^, SK^67^, EK^209^ | AK^98,^ ^123,^ ^316,^ ^368^, VK^175,^ ^302,^ ^398^, IK^57^, YK^348,^ ^420^, TK^149,^ ^318,^ ^388^, PK^246,^ ^274,^ ^276^, DK^241,^ ^250,^ ^442^, SK^76,^ ^161,^ ^366, 437^, EK^362^, QK^467^ | AK↓, VK↓, LK↓, IK↓, FK↓, WK↓, YK↓, TK↓, MK↓, PK↓, DK↓, SK↓, EK↓, QK↓ |  |
|  | TH^202^, SH^193^ | VH^196,^ ^313^, LH^338,^ ^461^, YH^60^, TH^252^, MH^35,^ ^457^, SH^53,^ ^296^ | AH↓, VH↓, LH↓, IH↓, FH↓, WH↓, YH↓, TH↓, MH↓, PH↓, DH↓, SH↓, EH↓, QH↓ |  |
|  | VE^165,^ ^208^, YE^51^, TE^215^, PE^188^, DE^84^, SE^128^, EE^129^ | AE^62^, VE^6,^ ^244,^ ^408^, LE^46^, IE^361^, WE^410^, PE^89,^ ^180,^ ^261,^ ^416^, DE^104,^ ^385^, EE^107, 322^ | AE↓, VE↓, LE↓, IE↓, FE↓, WE↓, YE↓, TE↓, ME↓, PE↓, DE↓, SE↓, EE↓, QE↓ |  |
|  | AN^133^, SN^28,^ ^174^ | VN^231^, LN^343^, FN^304^, WN^187^, YN^325^, MN^84^, DN^74,^ ^134^, SN^236,^ ^353,^ ^412^, EN^417^ | AN↓, VN↓, LN↓, IN↓, FN↓, WN↓, YN↓, TN↓, MN↓, PN↓, DN↓, SN↓, EN↓, QN↓ |  |
|  | LQ^80, 131^, YQ^38^, TQ^6,^ ^107^, PQ^46^, EQ^189^, QQ^39^ | VQ^3,^ ^13^, LQ^82,^ ^203^, WQ^446^, TQ^224,^ ^466^ , SQ26, EQ^323^, QQ^447^ | AQ↓, VQ↓, LQ↓, IQ↓, FQ↓, WQ↓, YQ↓, TQ↓, MQ↓, PQ↓, DQ↓, SQ↓, EQ↓, QQ↓ |  |
|  | **AS^66,^ ^95,^ ^180^, VS^11,^ ^35^, LS^97,^ ^184^, IS^21,^ ^142^, FS^64^, YS^197^, TS^27,^ ^71^, PS^9,^ ^57,^ ^126,^ ^170^, DS^157^, SS^98,^ ^127,^ ^158,^ ^181^, QS^173^** | **AS^25,^ ^50,^ ^147^, VS^144,^ ^185,^ ^295,^ ^352,^ ^392^, LS^21,^ ^211,^ ^470,^ ^472^, IS^52,^ ^71,^ ^282, 365^, FS^452^, WS^110^, YS^209,^ ^436^, TS^17,^ ^164,^ ^193^, PS^159,^ ^218,^ ^235,^ ^382,^ ^403^, DS^428^, SS^145,^ ^160,^ ^205,^ ^212,^ ^219,^ ^220^, ES^7,^ ^411^, QS^204^** | AS↓, VS↓, LS↓, IS↓, FS↓, WS↓, YS↓, TS↓, MS↓, PS↓, DS↓, SS↓, ES↓, QS↓ |  |
|  | AT^91,^ ^136^, VT^19,^ ^121,^ ^150,^ ^201^, LT^5,^ ^186^, IT^77^, TT^167,^ ^168^, PT^214^, ET^52,^ ^166^, QT^81^ | VT^288^, LT^192,^ ^387,^ ^394^, FT^69^, YT^378,^ ^465^, TT^422^, DT^91,^ ^278^, ST^148, 163,^ ^327^, QT^225^ | AT↓, VT↓, LT↓, IT↓, FT↓, WT↓, YT↓, TT↓, MT↓, PT↓, DT↓, ST↓, ET↓, QT↓ |  |
|  | LG^112^, IG^30^, FG^103^, TG^78,^ ^82^, TG^105^, PG^15,^ ^42,^ ^147^, SG^24,^ ^58,^ ^69,^ ^72^, EG^204^ | VG^130^, LG^171, 222, 264^, FG^116^, WG^66,^ ^136^, YG^33^, PG^15,^ ^42,^ ^474^, DG^31,^ ^55,^ ^430^, SG^8,^ ^165,^ ^189,^ ^206^, QG^138,^ ^448^ | AG↓, VG↓, LG↓, IG↓, FG↓, WG↓, YG↓, TG↓, MG↓, PG↓, DG↓, SG↓, EG↓, QG↓ |  |
|  | VF^102^, LF^123^, DF^144^ | VF^154,^ ^269,^ ^451^, LF^271^, FF^433^, YF^178^, TF^198^, DF^115^, SF^432^, QF^27^ | AF↓, VF↓, LF↓, IF↓, FF↓, WF↓, YF↓, TF↓, MF↓, PF↓, DF↓, SF↓, EF↓, QF↓ |  |
|  | AW^153^, TW^92^, SW^36^, QW^190^ | VW^65^, WW^109^, DW^341^, SW^186^, EW^108,^ ^409^ | AW↓, VW↓, LW↓, IW↓, FW↓, WW↓, YW↓, TW↓, MW↓, PW↓, DW↓, SW↓, EW↓, QW↓ |  |
|  | IY^50^, FY^145^, WY^37^, YY^88^, DY^87^, SY^182,^ ^196^ | VY^377^, LY^80,^ ^94,^ ^208,^ ^435^, FY^401^, YY^95,^ ^113^, TY^226, 328^, DY^112,^ ^177^, EY^347^, QY^324^ | AY↓, VY↓, LY↓, IY↓, FY↓, WY↓, YY↓, TY↓, MY↓, PY↓, DY↓, SY↓, EY↓, QY↓ |  |
|  | **VL^4,^ ^111^, LL^48^, YL^183^, TL^74,^ ^122^, SL^96,^ ^185^, EL^130^, QL^47^** | **AL^93,^ ^170,^ ^191,^ ^356,^ ^460^, VL^202,^ ^334,^ ^337,^ ^426^, FL^270,^ ^434^, WL^342^, YL^81^, TL^79,^ ^279,^ ^379^, PL^156^, DL^100^, SL^18,^ ^86,^ ^210,^ ^221,^ ^393,^ ^469,^ ^471^, EL^262,^ ^386^, QL^119^** | AL↓, VL↓, LL↓, IL↓, FL↓, WL↓, YL↓, TL↓, ML↓, PL↓, DL↓, SL↓, EL↓, QL↓ |  |
|  | Does not cleave | **VM^456^, LM^280^, QM^83^** | AM↓, VM↓, LM↓, IM↓, FM↓, WM↓, YM↓, TM↓, MM↓, PM↓, DM↓, SM↓, EM↓, QM↓ |  |
| **Cathepsin L** | **KR^55^, KQ^172^, KS^68,^ ^192^, KT^210^, KY^177^, KA^116,^ ^135,^ ^155,^ ^162^** | **KK^58^, KR^242^, KE^346^, KN^77,^ ^389^, KQ^118^, KS^124,^ ^162,^ ^247,^ ^443,^ ^468^, KT^251,^ ^317,^ ^363,^ ^421^, KG^44,^ ^150,^ ^369,^ ^399^, KF^303^, KY^59^, KA^355,^ ^367^, KL^438^** | KK↓, KR↓, KH↓, KE↓, KN↓, KQ↓, KS↓, KT↓, KG↓, KF↓, KW↓, KY↓, KA↓, KL↓, KM↓, | - *Generally the same as papain with these additional specificities* - *Exceptions at the P2 position with respect to papain are EX­_aa_ ,QX_aa_ , TX_aa_ , AX_aa_ where Cathepsin-L does not have specificity for* |
|  | WK^154^, WK^191^, WY^37^, WA^93^ | WE^410^, WN^187^, WQ^446^, WS^110^, WG^66,^ ^136^, WW^109^, WY^306^, WL^342^ | WK↓, WR↓, WH↓, WE↓, WN↓, WQ↓, WS↓, WT↓, WG↓, WF↓, WW↓, WY↓, WA↓, WL↓, WM↓ |  |
|  | HR^194^, HE^203^ | HK^233^, HE^297,^ ^458^, HN^314,^ ^462^, HQ^339^, HT^197,^ ^253^, HW^36^, HY^464^, HA^61^ | HK↓, HR↓, HH↓, HE↓, HN↓, HQ↓, HS↓, HT↓, HG↓, HF↓, HW↓, HY↓, HA↓, HL↓, HM↓ |  |
|  | VI^109^ , LI^49^ , LI^141^ | DI^405^, SI^51^, PI^360^, YI^227^, MI^281^ | KI↓, HI↓, DI↓, SI↓, PI↓, FI↓, WI↓, YI↓, VI↓, II↓, LI↓, MI↓ |  |
|  | **KV^18^, SV^3,^ ^10,^ ^120^, PV^160^, FV^34^, IV^110^, LV^138^** | **KV^64,^ ^239,^ ^351^, DV^294^, SV^153,^ ^213,^ ^268,^ ^333,^ ^455^, PV^182,^ ^425^, WV^37,^ ^48^, YV^307^, VV^12,^ ^214,^ ^291,^ ^292,^ ^331^, LV^5,^ ^129,^ ^174,^ ^397^, MV^141^** | KV↓, HV↓, DV↓, SV↓, PV↓, FV↓, WV↓, YV↓, VV↓, IV↓, LV↓, MV↓ |  |
| **Legumain** | **AN^133^** | **LN^343^** | AAN↓, AN↓, TN↓, LN↓ | Based on optimal sequences for schistosome (C197, N197C) and human legumains, and cruzain (Mathieu et al., 2002) |

Supplementary table S2| Sequencing primers which spanned the sgRNA region in the plant vector, pICH86966::AtU6p::sgRNA_PDS

| **Primer name** | **Sequence** |
| --- | --- |
| **newpsgRNAF** | TTCTGAGCGGGTCTGGATCT |
| **newpsgRNAR** | CGGTCACATGTGCATCCTCT |

Supplementary table S3| Agroinfiltration sample combinations which were analysed by SDS-PAGE for their effectiveness at disrupting *Nb*CysP6, *Nb*VPE1a and *Nb*VPE1b

|  | **Sample combinations for agroinfiltration** |
| --- | --- |
| Control samples | Cas9 + sgRNA:*Nb*CysP6 |
|  | Cas9 + sgRNA:*Nb*VPE1a/b |
|  | Cas9 + sgRNA:*Nb*CysP6 + sgRNA:*Nb*VPE1a/b |
| Experimental samples | CAP256-VRC26.25 heavy and light chains + hTPST1 |
|  | CAP256-VRC26.25 heavy and light chains + hTPST1 + Cas9 + sgRNA:*Nb*CysP6 |
|  | CAP256-VRC26.25 heavy and light chains + hTPST1 + Cas9 + sgRNA:*Nb*VPE1a/b |
|  | CAP256-VRC26.25 heavy and light chains + hTPST1 + Cas9 + sgRNA:*Nb*CysP6 + sgRNA:*Nb*VPE1a/b |

Supplementary table S4| Primers which span the *Nb*CysP6, *Nb*VPE1a and *Nb*VPE1b protease gene regions 250 bp on either side of the gene editing site

| **Primer name** | | **Sequence** |
| --- | --- | --- |
| **GECYSF** | GCTTCAATGGCAATGCTCACA | |
| **GECYSR** | ATGAACCAGGGAGTTGTTGGG | |
| **GEVPEF** | AGCCGAGGTTCGATTGGAAA | |
| **GEVPER** | GCAGAAGATCTGCATCACGC | |

Supplementary table S5| Primer sets to amplify reference and candidate genes for reverse transcription

|  | **Genbank Accession Number** | **Name** |  | **5’ – 3’** | **Amplicon size** | ***Tm (°C)** | **GC%** |
| --- | --- | --- | --- | --- | --- | --- | --- |
| Reference genes | XM_009790698.1^a^ | F-Box | Forward primer | CCTCCCAGGTACTTTCGTGC | 99 | 60.39 | 60 |
|  |  |  | Reverse primer | GCCTTGGTTCCAGGATGTGA |  | 59.96 | 55 |
|  | XM_009768212.1^b^ | PP2A | Forward primer | GCTAGCAGAGGACAGGCATT | 116 | 59.82 | 55 |
|  |  |  | Reverse primer | GCATACAAAGGGCACCAAGC |  | 60.11 | 55 |
| Target genes | KX375796.1^c^ | *Nb*CysP6 | Forward primer | TCTCCAATCAAGCCACCTAC | 113 | 60.39 | 55 |
|  |  |  | Reverse primer | GGGCAACATCCCCAAGAGAA |  | 59.96 | 55 |
|  | AB181187.1^d^ | *Nb*VPE1a | Forward primer | CTGCTGTTAGTGGAGGCAGT | 106 | 59.68 | 55 |
|  |  |  | Reverse primer | GTAGGCATCCCAAGCACTCC |  | 60.46 | 60 |
|  | AB181188.1^e^ | *Nb*VPE1b | Forward primer | CTTAGTGGAGGCAGCGGAAA | 112 | 60.04 | 55 |
|  |  |  | Reverse primer | GGTACGGATCGGTAGGCATC |  | 59.76 | 60 |

^a^PREDICTED: *Nicotiana sylvestris* F-box/kelch-repeat protein At5g15710 (LOC104236713), mRNA

^b^PREDICTED: *Nicotiana sylvestris* serine/threonine-protein phosphatase 2A 65 kDa regulatory subunit A beta isoform (LOC104217882), mRNA

^c^*Nicotiana benthamiana* papain-like cysteine proteinase 6 mRNA, complete cds

^d^*Nicotiana benthamiana* *Nb*VPE-1a mRNA for vacuolar processing enzyme 1a, partial cds

^e^*Nicotiana benthamiana* *Nb*VPE-1b mRNA for vacuolar processing enzyme 1b, partial cds

Supplementary table S6| Summary of statistical differences between cathepsin L-like protease activities when comparing the effects of cysteine protease disruption on cathepsin L-like protease activity against samples without CAP256-VRC26.25 expression

|  | **Negative control** | | | **LBA4404 (3dpi)** | | | **LBA4404 (7dpi)** | | | | **Cas9 + sgRNA:NbCysP6 (3dpi)** | | | **Cas9 + sgRNA:NbCysP6 (7dpi)** | **Cas9 + sgRNA:NbVPE1a/b (3dpi)** | | | **Cas9 + sgRNA:NbVPE1a/b (7dpi)** | **Cas9 + sgRNA:NbCysP6 + sgRNA:NbVPE1a/b (3dpi)** | | | **Cas9 + sgRNA:NbCysP6 + sgRNA:NbVPE1a/b (7dpi)** | | |
| --- | --- | --- | --- | --- | --- | --- | --- | --- | --- | --- | --- | --- | --- | --- | --- | --- | --- | --- | --- | --- | --- | --- | --- | --- |
| **Negative control** | **N/A** | | |  |  |  | | **89** | **5319** | ***** |  |  |  |  |  |  |  |  | **89** | **141** | ***** |  |  |  |
| **LBA4404 (3dpi)** |  |  |  | **N/A** | | | **473** | | **5319** | ***** |  |  |  |  |  |  |  |  |  |  |  |  |  |  |
| **LBA4404 (7dpi)** | **5319** | **89** | ***** | **5319** | **473** | ***** | | **N/A** | | | **5319** | **215** | ***** |  | **5319** | **286** | ***** |  | **5319** | **141** | ***** | **5319** | **576** | ***** |
| **Cas9 + sgRNA:NbCysP6 (3dpi)** |  |  |  |  |  |  | | **215** | **5319** | ***** | **N/A** | | |  |  |  |  |  |  |  |  |  |  |  |
| **Cas9 + sgRNA:NbCysP6 (7dpi)** |  |  |  |  |  |  | |  |  |  |  |  |  | **N/A** |  |  |  |  |  |  |  |  |  |  |
| **Cas9 + sgRNA:NbVPE1a/b (3dpi)** |  |  |  |  |  |  | | **286** | **5319** | ***** |  |  |  |  | **N/A** | | |  |  |  |  |  |  |  |
| **Cas9 + sgRNA:NbVPE1a/b (7dpi)** |  |  |  |  |  |  | |  |  |  |  |  |  |  |  |  |  | **N/A** |  |  |  |  |  |  |
| **Cas9 + sgRNA:NbCysP6 + sgRNA:NbVPE1a/b (3dpi)** | **141** | **89** | ***** |  |  |  | | **141** | **5319** | ***** |  |  |  |  |  |  |  |  | **N/A** | | |  |  |  |
| **Cas9 + sgRNA:NbCysP6 + sgRNA:NbVPE1a/b (7dpi)** |  |  |  |  |  |  | | **576** | **5319** | ***** |  |  |  |  |  |  |  |  |  |  |  | **N/A** | | |
| **CAP256-VRC26.25 (3dpi)** | **238** | **89** | ***** |  |  |  | | **238** | **5319** | ***** |  |  |  |  |  |  |  |  | **238** | **141** | ***** |  |  |  |
| **CAP256-VRC26.25 (7dpi)** | **332** | **89** | ****** |  |  |  | | **332** | **5319** | ***** |  |  |  |  |  |  |  |  | **332** | **141** | ****** |  |  |  |
| **CAP256-VRC26.25 + Cas9 + sgRNA:NbCysP6 (3dpi)** |  |  |  |  |  |  | | **121** | **5319** | ***** |  |  |  |  |  |  |  |  |  |  |  |  |  |  |
| **CAP256-VRC26.25 + Cas9 + sgRNA:NbCysP6 (7dpi)** | **134** | **89** | ***** |  |  |  | | **134** | **5319** | ***** |  |  |  |  |  |  |  |  |  |  |  |  |  |  |
| **CAP256-VRC26.25 + Cas9 + sgRNA:NbVPE1a/b (3dpi)** |  |  |  |  |  |  | | **252** | **5319** | ***** |  |  |  |  |  |  |  |  |  |  |  |  |  |  |
| **CAP256-VRC26.25 + Cas9 + sgRNA:NbVPE1a/b (7dpi)** | **362** | **89** | ****** |  |  |  | | **362** | **5319** | ***** |  |  |  |  |  |  |  |  | **362** | **141** | ****** |  |  |  |
| **CAP256-VRC26.25 + Cas9 + sgRNA:NbCysP6 + sgRNA:NbVPE1a/b (3dpi)** |  |  |  |  |  |  | | **157** | **5319** | ***** |  |  |  |  |  |  |  |  |  |  |  |  |  |  |
| **CAP256-VRC26.25 + Cas9 + sgRNA:NbCysP6 + sgRNA:NbVPE1a/b (7dpi)** |  |  |  |  |  |  | | **210** | **5319** | ***** |  |  |  |  |  |  |  |  |  |  |  |  |  |  |

§Statistically significant differences between control and experimental treatments were determined by single-factor ANOVA with replication (p-value < 0.001) and post-hoc t-tests (p-value < 0.05)

* Significant difference

** Highly significant difference

Supplementary table S7| Summary of statistical differences between cathepsin L-like protease activities when comparing the effects of cysteine protease disruption on cathepsin L-like protease activity against samples with CAP256-VRC26.25 expression.

|  | **CAP256-VRC26.25 (3dpi)** | | | **CAP256-VRC26.25 (7dpi)** | | | **CAP256-VRC26.25 + Cas9 + sgRNA:NbCysP6 (3dpi)** | | | **CAP256-VRC26.25 + Cas9 + sgRNA:NbCysP6 (7dpi)** | | | **CAP256-VRC26.25 + Cas9 + sgRNA:NbVPE1a/b (3dpi)** | | | **CAP256-VRC26.25 + Cas9 + sgRNA:NbVPE1a/b (7dpi)** | | | **CAP256-VRC26.25 + Cas9 + sgRNA:NbCysP6 + sgRNA:NbVPE1a/b (3dpi)** | | | **CAP256-VRC26.25 + Cas9 + sgRNA:NbCysP6 + sgRNA:NbVPE1a/b (7dpi)** | | |
| --- | --- | --- | --- | --- | --- | --- | --- | --- | --- | --- | --- | --- | --- | --- | --- | --- | --- | --- | --- | --- | --- | --- | --- | --- |
| **Negative control** | **89** | **238** | ***** | **89** | **332** | ****** |  |  |  | **89** | **134** | ***** |  |  |  | **89** | **362** | ****** |  |  |  |  |  |  |
| **LBA4404 (3dpi)** |  |  |  |  |  |  |  |  |  |  |  |  |  |  |  |  |  |  |  |  |  |  |  |  |
| **LBA4404 (7dpi)** | **5319** | **238** | ***** | **5319** | **332** | ***** | **5319** | **121** | ***** | **5319** | **134** | ***** | **5319** | **252** | ***** | **5319** | **362** | ***** | **5319** | **157** | ***** | **5319** | **210** | ***** |
| **Cas9 + sgRNA:NbCysP6 (3dpi)** |  |  |  |  |  |  |  |  |  |  |  |  |  |  |  |  |  |  |  |  |  |  |  |  |
| **Cas9 + sgRNA:NbCysP6 (7dpi)** |  |  |  |  |  |  |  |  |  |  |  |  |  |  |  |  |  |  |  |  |  |  |  |  |
| **Cas9 + sgRNA:NbVPE1a/b (3dpi)** |  |  |  |  |  |  |  |  |  |  |  |  |  |  |  |  |  |  |  |  |  |  |  |  |
| **Cas9 + sgRNA:NbVPE1a/b (7dpi)** |  |  |  |  |  |  |  |  |  |  |  |  |  |  |  |  |  |  |  |  |  |  |  |  |
| **Cas9 + sgRNA:NbCysP6 + sgRNA:NbVPE1a/b (3dpi)** | **141** | **238** | ***** | **141** | **332** | ****** |  |  |  |  |  |  |  |  |  | **141** | **362** | ****** |  |  |  |  |  |  |
| **Cas9 + sgRNA:NbCysP6 + sgRNA:NbVPE1a/b (7dpi)** |  |  |  |  |  |  |  |  |  |  |  |  |  |  |  |  |  |  |  |  |  |  |  |  |
| **CAP256-VRC26.25 (3dpi)** | **N/A** | | | **238** | **332** | ***** | **238** | **121** | ***** | **238** | **134** | ***** |  |  |  | **238** | **362** | ***** |  |  |  |  |  |  |
| **CAP256-VRC26.25 (7dpi)** | **332** | **238** | ***** | **N/A** | | | **332** | **121** | ***** | **332** | **134** | ****** |  |  |  |  |  |  | **332** | **157** | ***** |  |  |  |
| **CAP256-VRC26.25 + Cas9 + sgRNA:NbCysP6 (3dpi)** | **121** | **238** | ***** | **121** | **332** | ***** | **N/A** | | |  |  |  |  |  |  | **121** | **362** | ***** |  |  |  |  |  |  |
| **CAP256-VRC26.25 + Cas9 + sgRNA:NbCysP6 (7dpi)** | **134** | **238** | ***** | **134** | **332** | ****** |  |  |  | **N/A** | | |  |  |  | **134** | **362** | ****** |  |  |  |  |  |  |
| **CAP256-VRC26.25 + Cas9 + sgRNA:NbVPE1a/b (3dpi)** |  |  |  |  |  |  |  |  |  |  |  |  | **N/A** | | |  |  |  |  |  |  |  |  |  |
| **CAP256-VRC26.25 + Cas9 + sgRNA:NbVPE1a/b (7dpi)** | **362** | **238** | ***** |  |  |  | **362** | **121** | ***** | **362** | **134** | ****** |  |  |  | **N/A** | | | **362** | **157** | ***** |  |  |  |
| **CAP256-VRC26.25 + Cas9 + sgRNA:NbCysP6 + sgRNA:NbVPE1a/b (3dpi)** |  |  |  | **157** | **332** | ***** |  |  |  |  |  |  |  |  |  | **157** | **362** | ***** | **N/A** | | |  |  |  |
| **CAP256-VRC26.25 + Cas9 + sgRNA:NbCysP6 + sgRNA:NbVPE1a/b (7dpi)** |  |  |  |  |  |  |  |  |  |  |  |  |  |  |  |  |  |  |  |  |  | **N/A** | | |

§Statistically significant differences between control and experimental treatments were determined by single-factor ANOVA with replication (p-value < 0.001) and post-hoc t-tests (p-value < 0.05)

* Significant difference

** Highly significant difference

Supplementary table S8| Summary of statistical differences between legumain protease activities when comparing the effects of cysteine protease disruption on legumain protease activity against samples without CAP256-VRC26.25 expression.

|  | **Negative control** | | | **LBA4404 (3dpi)** | **LBA4404 (7dpi)** | | | **Cas9 + sgRNA:NbCysP6 (3dpi)** | | | **Cas9 + sgRNA:NbCysP6 (7dpi)** | | | **Cas9 + sgRNA:NbVPE1a/b (3dpi)** | | | **Cas9 + sgRNA:NbVPE1a/b (7dpi)** | | | **Cas9 + sgRNA:NbCysP6 + sgRNA:NbVPE1a/b (3dpi)** | | | **Cas9 + sgRNA:NbCysP6 + sgRNA:NbVPE1a/b (7dpi)** | | |
| --- | --- | --- | --- | --- | --- | --- | --- | --- | --- | --- | --- | --- | --- | --- | --- | --- | --- | --- | --- | --- | --- | --- | --- | --- | --- |
| **Negative control** | **N/A** | | |  |  |  |  |  |  |  |  |  |  |  |  |  |  |  |  | **172** | **351** | ***** |  |  |  |
| **LBA4404 (3dpi)** |  |  |  | **N/A** |  |  |  |  |  |  |  |  |  |  |  |  |  |  |  |  |  |  |  |  |  |
| **LBA4404 (7dpi)** |  |  |  |  | **N/A** | | |  |  |  | **256** | **81** | ***** |  |  |  |  |  |  |  |  |  |  |  |  |
| **Cas9 + sgRNA:NbCysP6 (3dpi)** |  |  |  |  |  |  |  | **N/A** | | |  |  |  |  |  |  |  |  |  | **181** | **351** | ***** |  |  |  |
| **Cas9 + sgRNA:NbCysP6 (7dpi)** |  |  |  |  | **81** | **256** | ***** |  |  |  | **N/A** | | |  |  |  |  |  |  | **81** | **351** | ***** |  |  |  |
| **Cas9 + sgRNA:NbVPE1a/b (3dpi)** |  |  |  |  |  |  |  |  |  |  |  |  |  | **N/A** | | |  |  |  | **155** | **351** | ***** |  |  |  |
| **Cas9 + sgRNA:NbVPE1a/b (7dpi)** |  |  |  |  |  |  |  |  |  |  |  |  |  |  |  |  | **N/A** | | | **144** | **351** | ****** |  |  |  |
| **Cas9 + sgRNA:NbCysP6 + sgRNA:NbVPE1a/b (3dpi)** | **351** | **172** | ***** |  |  |  |  | **351** | **181** | ***** | **351** | **81** | ***** | **351** | **155** | ***** | **351** | **144** | ****** | **N/A** | | | **351** | **127** | ***** |
| **Cas9 + sgRNA:NbCysP6 + sgRNA:NbVPE1a/b (7dpi)** |  |  |  |  |  |  |  |  |  |  |  |  |  |  |  |  |  |  |  | **127** | **351** | ***** | **N/A** | | |
| **CAP256-VRC26.25 (3dpi)** |  |  |  |  |  |  |  |  |  |  | **265** | **81** | ***** |  |  |  | **265** | **144** | ***** |  |  |  | **265** | **127** | ***** |
| **CAP256-VRC26.25 (7dpi)** |  |  |  |  |  |  |  |  |  |  |  |  |  |  |  |  |  |  |  |  |  |  |  |  |  |
| **CAP256-VRC26.25 + Cas9 + sgRNA:NbCysP6 (3dpi)** |  |  |  |  |  |  |  |  |  |  | **263** | **81** | ***** |  |  |  |  |  |  |  |  |  |  |  |  |
| **CAP256-VRC26.25 + Cas9 + sgRNA:NbCysP6 (7dpi)** | **445** | **172** | ***** |  | **445** | **256** | ***** | **445** | **181** | ***** | **445** | **81** | ****** | **445** | **155** | ****** | **445** | **144** | ***** | **445** | **351** | ***** | **445** | **127** | ***** |
| **CAP256-VRC26.25 + Cas9 + sgRNA:NbVPE1a/b (3dpi)** |  |  |  |  |  |  |  |  |  |  |  |  |  |  |  |  |  |  |  |  |  |  |  |  |  |
| **CAP256-VRC26.25 + Cas9 + sgRNA:NbVPE1a/b (7dpi)** | **446** | **172** | ***** |  | **446** | **256** | ***** | **446** | **181** | ***** | **446** | **81** | ***** | **446** | **155** | ***** | **446** | **144** | ***** |  |  |  | **446** | **127** | ***** |
| **CAP256-VRC26.25 + Cas9 + sgRNA:NbCysP6 + sgRNA:NbVPE1a/b (3dpi)** | **318** | **172** | ***** |  |  |  |  | **318** | **181** | ***** | **318** | **81** | ***** | **318** | **155** | ***** | **318** | **144** | ***** |  |  |  | **318** | **127** | ***** |
| **CAP256-VRC26.25 + Cas9 + sgRNA:NbCysP6 + sgRNA:NbVPE1a/b (7dpi)** | **461** | **172** | ***** |  |  |  |  | **461** | **181** | ***** | **461** | **81** | ***** |  |  |  | **461** | **144** | ***** |  |  |  | **461** | **127** | ***** |

§Statistically significant differences between control and experimental treatments were determined by single-factor ANOVA with replication (p-value < 0.001) and post-hoc t-tests (p-value < 0.05)

* Significant difference

** Highly significant difference

Supplementary table S9| Summary of statistical differences between legumain protease activities when comparing the effects of cysteine protease disruption on legumain protease activity against samples with CAP256-VRC26.25 expression.

|  | **CAP256-VRC26.25 (3dpi)** | | | **CAP256-VRC26.25 (7dpi)** | **CAP256-VRC26.25 + Cas9 + sgRNA:NbCysP6 (3dpi)** | | | **CAP256-VRC26.25 + Cas9 + sgRNA:NbCysP6 (7dpi)** | | | **CAP256-VRC26.25 + Cas9 + sgRNA:NbVPE1a/b (3dpi)** | **CAP256-VRC26.25 + Cas9 + sgRNA:NbVPE1a/b (7dpi)** | | | **CAP256-VRC26.25 + Cas9 + sgRNA:NbCysP6 + sgRNA:NbVPE1a/b (3dpi)** | | | **CAP256-VRC26.25 + Cas9 + sgRNA:NbCysP6 + sgRNA:NbVPE1a/b (7dpi)** | | |
| --- | --- | --- | --- | --- | --- | --- | --- | --- | --- | --- | --- | --- | --- | --- | --- | --- | --- | --- | --- | --- |
| **Negative control** |  |  |  |  |  |  |  | **172** | **445** | ***** |  | **172** | **446** | ***** | **172** | **318** | ***** | **172** | **461** | ***** |
| **LBA4404 (3dpi)** |  |  |  |  |  |  |  |  |  |  |  |  |  |  |  |  |  |  |  |  |
| **LBA4404 (7dpi)** |  |  |  |  |  |  |  | **256** | **445** | ***** |  | **256** | **446** | ***** |  |  |  |  |  |  |
| **Cas9 + sgRNA:NbCysP6 (3dpi)** |  |  |  |  |  |  |  | **181** | **445** | ***** |  | **181** | **446** | ***** | **181** | **318** | ***** | **181** | **461** | ***** |
| **Cas9 + sgRNA:NbCysP6 (7dpi)** | **81** | **265** | ***** |  | **81** | **263** | ***** | **81** | **445** | ****** |  | **81** | **446** | ***** | **81** | **318** | ***** | **81** | **461** | ***** |
| **Cas9 + sgRNA:NbVPE1a/b (3dpi)** |  |  |  |  |  |  |  | **155** | **445** | ****** |  | **155** | **446** | ***** | **155** | **318** | ***** |  |  |  |
| **Cas9 + sgRNA:NbVPE1a/b (7dpi)** | **144** | **265** | ***** |  |  |  |  | **144** | **445** | ***** |  | **144** | **446** | ***** | **144** | **318** | ***** | **144** | **461** | ***** |
| **Cas9 + sgRNA:NbCysP6 + sgRNA:NbVPE1a/b (3dpi)** |  |  |  |  |  |  |  | **351** | **445** | ***** |  |  |  |  |  |  |  |  |  |  |
| **Cas9 + sgRNA:NbCysP6 + sgRNA:NbVPE1a/b (7dpi)** | **127** | **265** | ***** |  |  |  |  | **127** | **445** | ****** |  | **127** | **446** | ***** | **127** | **318** | ***** | **127** | **461** | ***** |
| **CAP256-VRC26.25 (3dpi)** | **N/A** | | |  |  |  |  | **265** | **445** | ***** |  | **265** | **446** | ***** |  |  |  |  |  |  |
| **CAP256-VRC26.25 (7dpi)** |  |  |  | **N/A** |  |  |  |  |  |  |  |  |  |  |  |  |  |  |  |  |
| **CAP256-VRC26.25 + Cas9 + sgRNA:NbCysP6 (3dpi)** |  |  |  |  | **N/A** | | | **263** | **445** | ***** |  | **263** | **446** | ***** |  |  |  |  |  |  |
| **CAP256-VRC26.25 + Cas9 + sgRNA:NbCysP6 (7dpi)** | **445** | **265** | ***** |  | **445** | **263** | ***** | **N/A** | | |  |  |  |  |  |  |  |  |  |  |
| **CAP256-VRC26.25 + Cas9 + sgRNA:NbVPE1a/b (3dpi)** |  |  |  |  |  |  |  |  |  |  | **N/A** |  |  |  |  |  |  |  |  |  |
| **CAP256-VRC26.25 + Cas9 + sgRNA:NbVPE1a/b (7dpi)** | **446** | **265** | ***** |  | **446** | **263** | ***** |  |  |  |  | **N/A** | | |  |  |  |  |  |  |
| **CAP256-VRC26.25 + Cas9 + sgRNA:NbCysP6 + sgRNA:NbVPE1a/b (3dpi)** |  |  |  |  |  |  |  |  |  |  |  |  |  |  | **N/A** | | |  |  |  |
| **CAP256-VRC26.25 + Cas9 + sgRNA:NbCysP6 + sgRNA:NbVPE1a/b (7dpi)** |  |  |  |  |  |  |  |  |  |  |  |  |  |  |  |  |  | **N/A** | | |

§Statistically significant differences between control and experimental treatments were determined by single-factor ANOVA with replication (p-value < 0.001) and post-hoc t-tests (p-value < 0.05)

* Significant difference

** Highly significant difference

Supplementary table S10| Summary of statistical differences between sample TSP when comparing the effects of protease disruption on TSP against samples without CAP256-VRC26.25 expression.

|  | **Negative control** | | | **LBA4404 (3dpi)** | | | **LBA4404 (7dpi)** | | | **Cas9 + sgRNA:NbCysP6 (3dpi)** | | | **Cas9 + sgRNA:NbCysP6 (7dpi)** | | | **Cas9 + sgRNA:NbVPE1a/b (3dpi)** | | | **Cas9 + sgRNA:NbVPE1a/b (7dpi)** | | | **Cas9 + sgRNA:NbCysP6 + sgRNA:NbVPE1a/b (3dpi)** | | | **Cas9 + sgRNA:NbCysP6 + sgRNA:NbVPE1a/b (7dpi)** | | |
| --- | --- | --- | --- | --- | --- | --- | --- | --- | --- | --- | --- | --- | --- | --- | --- | --- | --- | --- | --- | --- | --- | --- | --- | --- | --- | --- | --- |
| **Negative control** | **N/A** | | | **3.45** | **3.91** | ***** |  |  |  | **3.45** | **2.62** | ****** | **3.45** | **1.93** | ****** | **3.45** | **2.34** | ****** |  |  |  |  |  |  | **3.45** | **2.60** | ****** |
| **LBA4404 (3dpi)** |  |  |  | **N/A** | | | **3.91** | **2.74** | ***** | **3.91** | **2.62** | ***** | **3.91** | **1.93** | ***** | **3.91** | **2.34** | ***** | **3.91** | **2.09** | ***** | **3.91** | **3.12** | ***** | **3.91** | **2.60** | ***** |
| **LBA4404 (7dpi)** | **2.74** | **3.45** | ***** | **2.74** | **3.91** | ***** | **N/A** | | |  |  |  | **2.74** | **1.93** | ***** |  |  |  | **2.74** | **2.09** | ***** |  |  |  |  |  |  |
| **Cas9 + sgRNA:NbCysP6 (3dpi)** | **2.62** | **3.45** | ****** | **2.62** | **3.91** | ***** |  |  |  | **N/A** | | | **2.62** | **1.93** | ***** | **2.62** | **2.34** | ***** | **2.62** | **2.09** | ***** |  |  |  |  |  |  |
| **Cas9 + sgRNA:NbCysP6 (7dpi)** | **1.93** | **3.45** | ****** | **1.93** | **3.91** | ***** | **1.93** | **2.74** | ***** | **1.93** | **2.62** | ***** | **N/A** | | | **1.93** | **2.34** | ***** |  |  |  | **1.93** | **3.12** | ***** | **1.93** | **2.60** | ***** |
| **Cas9 + sgRNA:NbVPE1a/b (3dpi)** | **2.34** | **3.45** | ****** | **2.34** | **3.91** | ***** |  |  |  | **2.34** | **2.62** | ***** | **2.34** | **1.93** | ***** | **N/A** | | | **2.34** | **2.09** | ***** | **2.34** | **3.12** | ***** | **2.34** | **2.60** | ***** |
| **Cas9 + sgRNA:NbVPE1a/b (7dpi)** | **2.09** | **3.45** | ****** | **2.09** | **3.91** | ***** | **2.09** | **2.74** | ***** | **2.09** | **2.62** | ***** |  |  |  | **2.09** | **2.34** | ***** | **N/A** | | | **2.09** | **3.12** | ***** | **2.09** | **2.60** | ***** |
| **Cas9 + sgRNA:NbCysP6 + sgRNA:NbVPE1a/b (3dpi)** |  |  |  | **3.12** | **3.91** | ***** |  |  |  |  |  |  | **3.12** | **1.93** | ***** | **3.12** | **2.34** | ***** | **3.12** | **2.09** | ***** | **N/A** | | |  |  |  |
| **Cas9 + sgRNA:NbCysP6 + sgRNA:NbVPE1a/b (7dpi)** | **2.60** | **3.45** | ****** | **2.60** | **3.91** | ***** |  |  |  | **2.60** | **2.62** | ***** | **2.60** | **1.93** | ***** | **2.60** | **2.34** | ***** |  |  |  |  |  |  | **N/A** | | |
| **CAP256-VRC26.25 (3dpi)** | **2.36** | **3.45** | ***** | **2.36** | **3.91** | ***** | **2.36** | **2.74** | ***** |  |  |  | **2.36** | **1.93** | ***** |  |  |  |  |  |  | **2.36** | **3.12** | ***** |  |  |  |
| **CAP256-VRC26.25 (7dpi)** | **1.82** | **3.45** | ****** | **1.82** | **3.91** | ***** | **1.82** | **2.74** | ***** | **1.82** | **2.62** | ****** |  |  |  | **1.82** | **2.34** | ***** |  |  |  | **1.82** | **3.12** | ***** | **1.82** | **2.60** | ***** |
| **CAP256-VRC26.25 + Cas9 + sgRNA:NbCysP6 (3dpi)** | **4.54** | **3.45** | ***** | **4.54** | **3.91** | ***** | **4.54** | **2.74** | ****** | **4.54** | **2.62** | ****** | **4.54** | **1.93** | ****** | **4.54** | **2.34** | ***** | **4.54** | **2.09** | ***** | **4.54** | **3.12** | ***** | **4.54** | **2.60** | ****** |
| **CAP256-VRC26.25 + Cas9 + sgRNA:NbCysP6 (7dpi)** | **4.01** | **3.45** | ***** |  |  |  | **4.01** | **2.74** | ***** | **4.01** | **2.62** | ****** | **4.01** | **1.93** | ****** | **4.01** | **2.34** | ****** | **4.01** | **2.09** | ***** | **4.01** | **3.12** | ***** | **4.01** | **2.60** | ****** |
| **CAP256-VRC26.25 + Cas9 + sgRNA:NbVPE1a/b (3dpi)** | **2.69** | **3.45** | ***** | **2.69** | **3.91** | ***** |  |  |  |  |  |  | **2.69** | **1.93** | ***** |  |  |  | **2.69** | **2.09** | ***** |  |  |  |  |  |  |
| **CAP256-VRC26.25 + Cas9 + sgRNA:NbVPE1a/b (7dpi)** | **2.28** | **3.45** | ****** | **2.28** | **3.91** | ***** | **2.28** | **2.74** | ***** | **2.28** | **2.62** | ***** | **2.28** | **1.93** | ***** |  |  |  | **2.28** | **2.09** | ***** | **2.28** | **3.12** | ***** | **2.28** | **2.60** | ***** |
| **CAP256-VRC26.25 + Cas9 + sgRNA:NbCysP6 + sgRNA:NbVPE1a/b (3dpi)** | **2.43** | **3.45** | ***** | **2.43** | **3.91** | ***** |  |  |  |  |  |  | **2.43** | **1.93** | ***** |  |  |  | **2.43** | **2.09** | ***** | **2.43** | **3.12** | ***** |  |  |  |
| **CAP256-VRC26.25 + Cas9 + sgRNA:NbCysP6 + sgRNA:NbVPE1a/b (7dpi)** | **1.79** | **3.45** | ***** | **1.79** | **3.91** | ***** |  |  |  |  |  |  |  |  |  |  |  |  |  |  |  | **1.79** | **3.12** | ***** |  |  |  |

§Statistically significant differences between control and experimental treatments were determined by single-factor ANOVA with replication (p-value < 0.001) and post-hoc t-tests (p-value < 0.05)

* Significant difference

** Highly significant difference

Supplementary table S11| Summary of statistical differences between sample TSP when comparing the effects of protease disruption on TSP against samples with CAP256-VRC26.25 expression.

|  | **CAP256-VRC26.25 (3dpi** | | | **CAP256-VRC26.25 (7dpi)** | | | **CAP256-VRC26.25 + Cas9 + sgRNA:NbCysP6 (3dpi)** | | | **CAP256-VRC26.25 + Cas9 + sgRNA:NbCysP6 (7dpi)** | | | **CAP256-VRC26.25 + Cas9 + sgRNA:NbVPE1a/b (3dpi)** | | | **CAP256-VRC26.25 + Cas9 + sgRNA:NbVPE1a/b (7dpi)** | | | **CAP256-VRC26.25 + Cas9 + sgRNA:NbCysP6 + sgRNA:NbVPE1a/b (3dpi)** | | | **CAP256-VRC26.25 + Cas9 + sgRNA:NbCysP6 + sgRNA:NbVPE1a/b (7dpi)** | | |
| --- | --- | --- | --- | --- | --- | --- | --- | --- | --- | --- | --- | --- | --- | --- | --- | --- | --- | --- | --- | --- | --- | --- | --- | --- |
| **Negative control** | **3.45** | **2.36** | ***** | **3.45** | **1.82** | ****** | **3.45** | **4.54** | ***** | **3.45** | **4.01** | ***** | **3.45** | **2.69** | ***** | **3.45** | **2.28** | ****** | **3.45** | **2.43** | ***** | **3.45** | **1.79** | ***** |
| **LBA4404 (3dpi)** | **3.91** | **2.36** | ***** | **3.91** | **1.82** | ***** | **3.91** | **4.54** | ***** |  |  |  | **3.91** | **2.69** | ***** | **3.91** | **2.28** | ***** | **3.91** | **2.43** | ***** | **3.91** | **1.79** | ***** |
| **LBA4404 (7dpi)** | **2.74** | **2.36** | ***** | **2.74** | **1.82** | ***** | **2.74** | **4.54** | ****** | **2.74** | **4.01** | ***** |  |  |  | **2.74** | **2.28** | ***** |  |  |  |  |  |  |
| **Cas9 + sgRNA:NbCysP6 (3dpi)** |  |  |  | **2.62** | **1.82** | ****** | **2.62** | **4.54** | ****** | **2.62** | **4.01** | ****** |  |  |  | **2.62** | **2.28** | ***** |  |  |  |  |  |  |
| **Cas9 + sgRNA:NbCysP6 (7dpi)** | **1.93** | **2.36** | ***** |  |  |  | **1.93** | **4.54** | ****** | **1.93** | **4.01** | ****** | **1.93** | **2.69** | ***** | **1.93** | **2.28** | ***** | **1.93** | **2.43** | ***** |  |  |  |
| **Cas9 + sgRNA:NbVPE1a/b (3dpi)** |  |  |  | **2.34** | **1.82** | ***** | **2.34** | **4.54** | ***** | **2.34** | **4.01** | ****** | **2.34** | **2.69** | ***** |  |  |  |  |  |  |  |  |  |
| **Cas9 + sgRNA:NbVPE1a/b (7dpi)** |  |  |  |  |  |  | **2.09** | **4.54** | ***** | **2.09** | **4.01** | ***** | **2.09** | **2.69** | ***** | **2.09** | **2.28** | ***** | **2.09** | **2.43** | ***** |  |  |  |
| **Cas9 + sgRNA:NbCysP6 + sgRNA:NbVPE1a/b (3dpi)** | **3.12** | **2.36** | ***** | **3.12** | **1.82** | ***** | **3.12** | **4.54** | ***** | **3.12** | **4.01** | ***** | **3.12** | **2.69** | ***** | **3.12** | **2.28** | ***** | **3.12** | **2.43** | ***** | **3.12** | **1.79** | ***** |
| **Cas9 + sgRNA:NbCysP6 + sgRNA:NbVPE1a/b (7dpi)** |  |  |  | **2.60** | **1.82** | ***** | **2.60** | **4.54** | ****** | **2.60** | **4.01** | ****** |  |  |  | **2.60** | **2.28** | ***** |  |  |  |  |  |  |
| **CAP256-VRC26.25 (3dpi)** | **N/A** | | | **2.36** | **1.82** | ***** | **2.36** | **4.54** | ****** | **2.36** | **4.01** | ****** | **2.36** | **2.69** | ***** |  |  |  |  |  |  |  |  |  |
| **CAP256-VRC26.25 (7dpi)** | **1.82** | **2.36** | ***** | **N/A** | | | **1.82** | **4.54** | ****** | **1.82** | **4.01** | ****** | **1.82** | **2.69** | ***** | **1.82** | **2.28** | ***** | **1.82** | **2.43** | ***** |  |  |  |
| **CAP256-VRC26.25 + Cas9 + sgRNA:NbCysP6 (3dpi)** | **4.54** | **2.36** | ****** | **4.54** | **1.82** | ****** | **N/A** | | | **4.54** | **4.01** | ***** | **4.54** | **2.69** | ****** | **4.54** | **2.28** | ****** | **4.54** | **2.43** | ****** | **4.54** | **1.79** | ***** |
| **CAP256-VRC26.25 + Cas9 + sgRNA:NbCysP6 (7dpi)** | **4.01** | **2.36** | ****** | **4.01** | **1.82** | ****** | **4.01** | **4.54** | ***** | **N/A** | | | **4.01** | **2.69** | ****** | **4.01** | **2.28** | ****** | **4.01** | **2.43** | ****** | **4.01** | **1.79** | ***** |
| **CAP256-VRC26.25 + Cas9 + sgRNA:NbVPE1a/b (3dpi)** | **2.69** | **2.36** | ***** | **2.69** | **1.82** | ***** | **2.69** | **4.54** | ****** | **2.69** | **4.01** | ****** | **N/A** | | | **2.69** | **2.28** | ***** |  |  |  |  |  |  |
| **CAP256-VRC26.25 + Cas9 + sgRNA:NbVPE1a/b (7dpi)** |  |  |  | **2.28** | **1.82** | ***** | **2.28** | **4.54** | ****** | **2.28** | **4.01** | ****** | **2.28** | **2.69** | ***** | **N/A** | | |  |  |  |  |  |  |
| **CAP256-VRC26.25 + Cas9 + sgRNA:NbCysP6 + sgRNA:NbVPE1a/b (3dpi)** |  |  |  | **2.43** | **1.82** | ***** | **2.43** | **4.54** | ****** | **2.43** | **4.01** | ****** |  |  |  |  |  |  | **N/A** | | |  |  |  |
| **CAP256-VRC26.25 + Cas9 + sgRNA:NbCysP6 + sgRNA:NbVPE1a/b (7dpi)** |  |  |  |  |  |  | **1.79** | **4.54** | ***** | **1.79** | **4.01** | ***** | **1.79** | **2.69** | ***** |  |  |  |  |  |  | **N/A** | | |

§Statistically significant differences between control and experimental treatments were determined by single-factor ANOVA with replication (p-value < 0.001) and post-hoc t-tests (p-value < 0.05)

* Significant difference

** Highly significant difference
