# Supplementary figures and images for: Transient proteolysis reduction of Nicotiana benthamiana-produced CAP256 broadly neutralizing antibodies using CRISPR/Cas9
Source: Front Plant Sci. 2022 Aug 18;13:953654. doi: 10.3389/fpls.2022.953654 (PMC9433777; doi:10.3389/fpls.2022.953654)

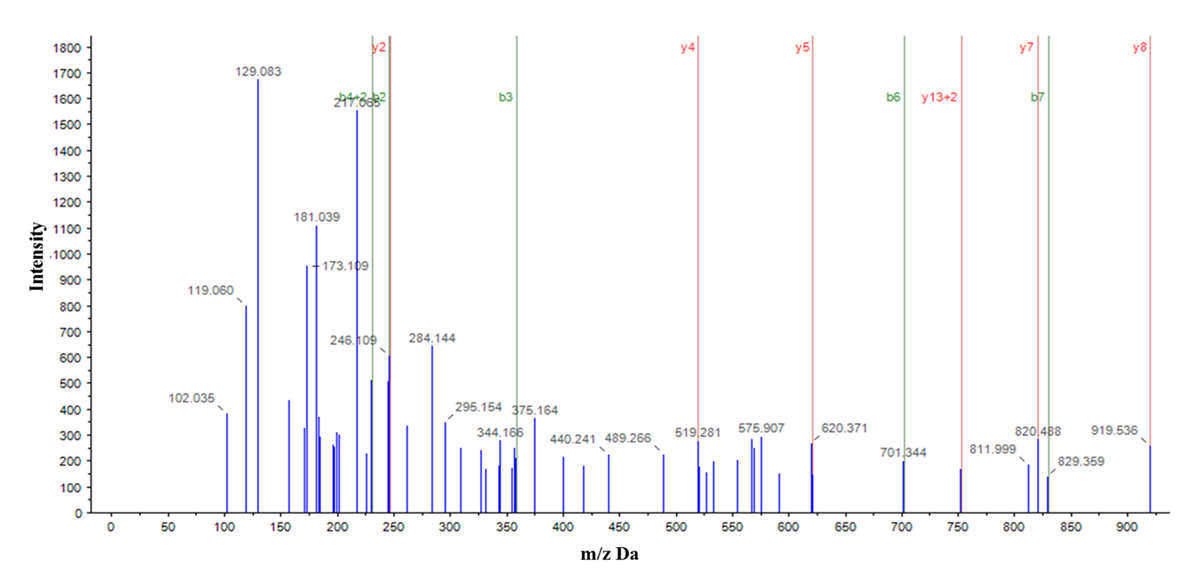

Supplement: Supplementary file 1 [file Data_Sheet_1.zip › Figure S1.TIF]

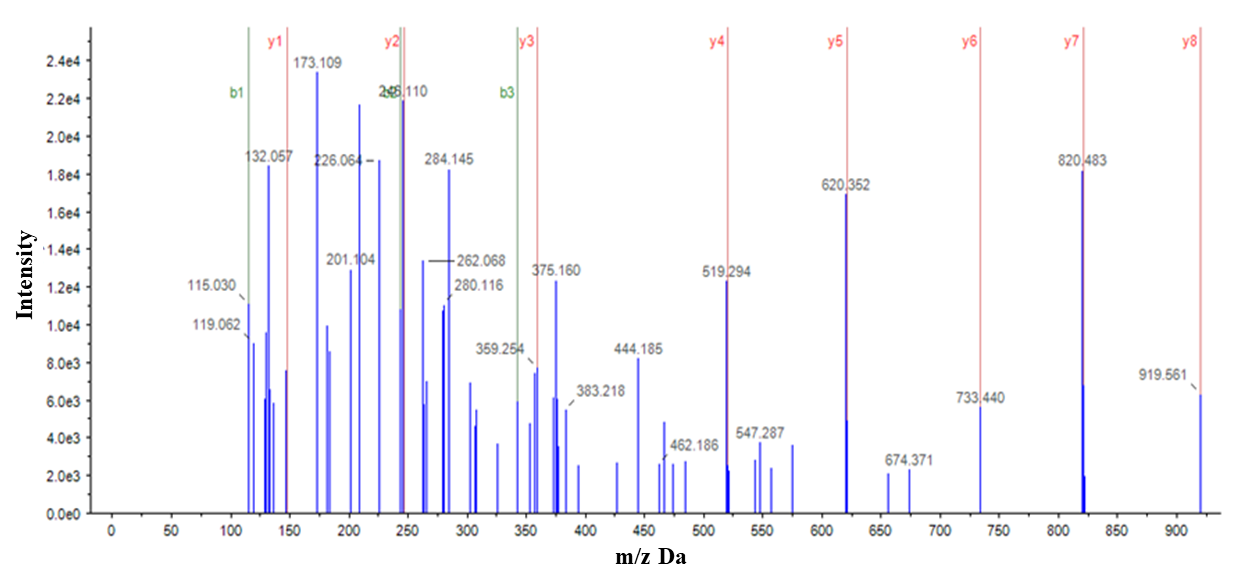

Supplement: Supplementary file 1 [file Data_Sheet_1.zip › Figure S2.TIF]

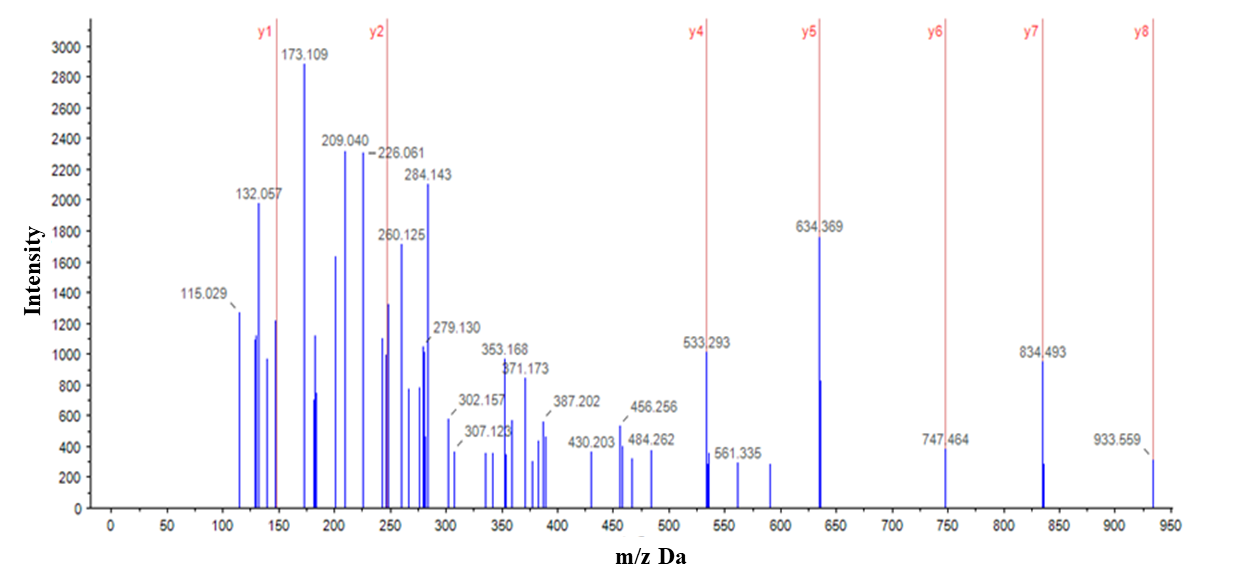

Supplement: Supplementary file 1 [file Data_Sheet_1.zip › Figure S3.TIF]

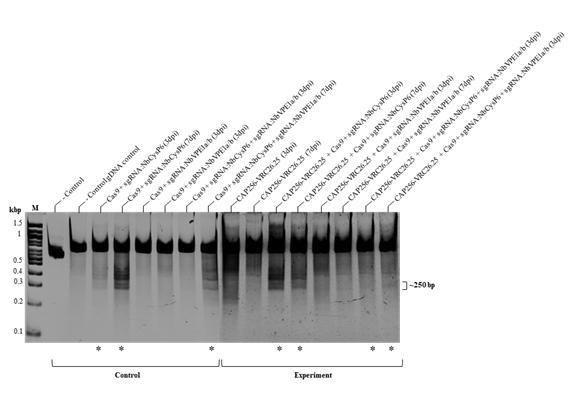

Supplement: Supplementary file 1 [file Data_Sheet_1.zip › Figure S4A.TIF]

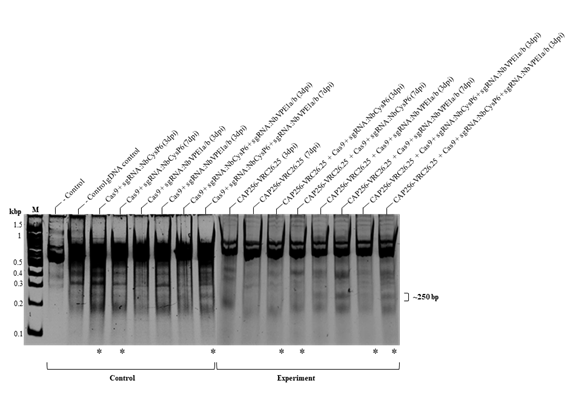

Supplement: Supplementary file 1 [file Data_Sheet_1.zip › Figure S4B.TIF]

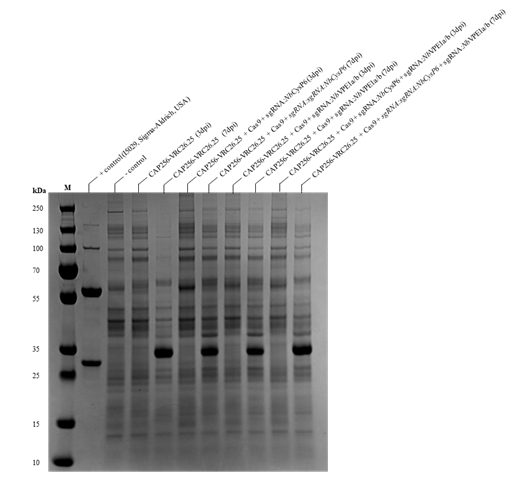

Supplement: Supplementary file 1 [file Data_Sheet_1.zip › Figure S5A.TIF]

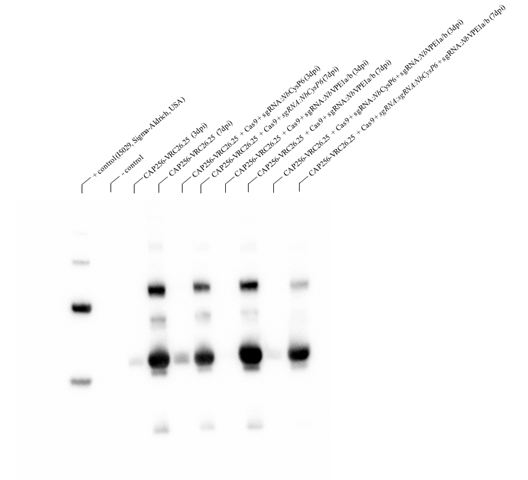

Supplement: Supplementary file 1 [file Data_Sheet_1.zip › Figure S5B.TIF]
